# Structural and electronic properties of Mo_6_S_3_I_6_ nanowires by newly proposed theoretical compositional ordering

**DOI:** 10.1038/s41598-018-37818-7

**Published:** 2019-02-04

**Authors:** You Kyoung Chung, Weon-Gyu Lee, Sudong Chae, Jae-Young Choi, Joonsuk Huh

**Affiliations:** 1 Department of Chemistry, Sungkyunkwan University, Suwon, 16419 Korea; 2 School of Advanced Materials Science & Engineering, Sungkyunkwan University, Suwon, 16419 Republic of Korea; 3 SKKU Advanced Institute of Nanotechnology (SAINT), Sungkyunkwan University, Suwon, 16419 Republic of Korea

## Abstract

The structural, electronic, and magnetic properties of molybdenum-based nanowires have been actively investigated for their potential applications in nanodevices; however, further advancement is hindered by incomplete knowledge of the electronic and atomic structures of Mo_6_S_3_I_6_. To facilitate further development of Mo_6_S_3_I_6_ nanowire devices, we propose possible atomic structures and corresponding electronic properties of Mo_6_S_3_I_6_ nanowires based on density functional theory. We explored various combinations of atomic structures by changing the positions of sulfur and iodine atoms linked to the two Mo_6_ octahedra in the Mo_6_S_3_I_6_ unit cell. We found two stable local energy minima structures characterized by elongation of the wire length, and therefore propose 28 possible atomic configurations. We calculated band structures of the newly proposed atomic models and found three structures that behaved as conductors. According to our compositional ordering structural analysis, we concluded that (i) periodic distortion of the bond lengths influences the behavior of the electrons in the system, (ii) the role of sulfur atoms in the bridging plane is important for intramolecular charge transport due to delocalized charge differences, and (iii) the electronic band gap energy is proportional to the integrated Mo-S bonding orbital energy.

## Introduction

The structural and electronic properties of one-dimensional materials such as LiMo_3_Se_3_, Mo_6_S_9−x_I_x_ have been widely investigated since molybdenum-based transition metal dichalcogenides (TMDCs) nanowires emerged in molecular electronics^1–8^. Unlike LiMo_3_Se_3_, which is composed of ionic bonds and decomposes rapidly in air, Mo_6_S_9−x_I_x_ can be prepared as a non-defective, uniform substance due to its air stability^9^ and the van der Waals interactions between its chains. Although the extraordinary stability of Mo_6_S_9−x_I_x_ is well known, the details of its structure that lead to this stability remain unknown.

In an early study of Mo_6_S_9−x_I_x_ nanowires, Milhailovic *et al*. revealed that Mo_6_S_3_I_6_ behaves as a quasi-one-dimensional conductor in the entire range of the study’s targeted strains^9,10^, and isomers of Mo_12_S_9_I_9_ were identified either as conductors or narrow-gap semiconductors^11^. Tománek *et al*. found Mo_6_S_3_I_6_ with sulfur atoms positioned in Mo-S-Mo bridges are particularly stable and identified Mo_6_S_4.5_I_4.5_ as a conductor^12^. An additional study regarding the effect of the inter-wire interaction showed that some particular isomers of bundled Mo_6_S_4.5_I_4.5_ and an isolated Mo_6_S_3_I_6_ nanowire are conductors^13^. In a later study by Muragan *et al*.^14^, the role of the valence electron concentration (VEC) on the structural stability and electronic properties of Mo_6_S_9−x_I_x_ nanowires was discussed, and Mo_6_S_7.5_I_1.5_ was reported as a conductor. However, the crystallographic structure of Mo_6_S_3_I_6_ nanowires is still uncertain because the positions of sulfur and iodine atoms have not been precisely determined by any experimental structural analysis methods such as field emission microscopy^15^ or x-ray diffraction^16^.

**Figure 1:**
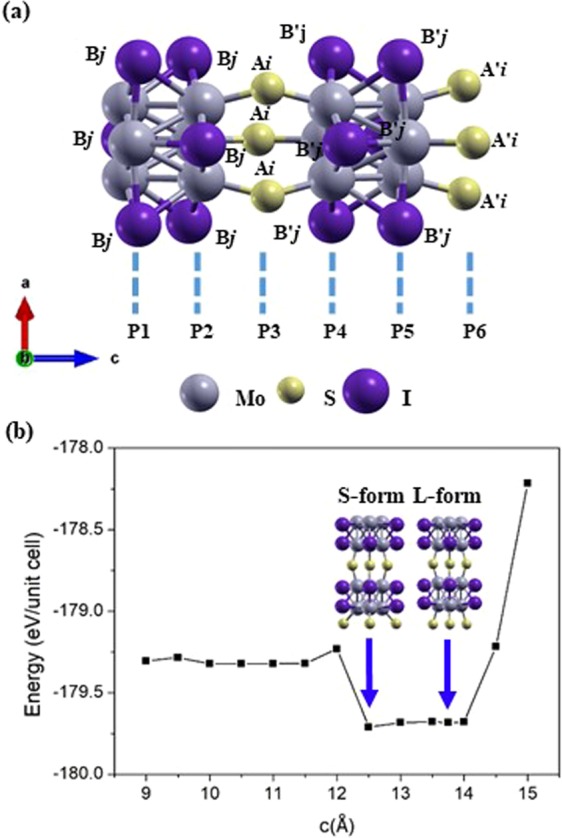
(**a**) Schematic atomic arrangement of the Mo_6_S_3_I_6_ nanowire unit cell used as a starting point for structural calculations. The unit cell contains 2 formula units, which includes 30 atoms arranged to form two Mo_6_ octahedra. The decorative and bridging sites of sulfur and iodine atoms linked to the two Mo_6_ octahedra in the unit cell are labeled as B*j*, B′*j* and A*i*, A′*i*, where *i* = 0–3 and *j* = 1–6. (**b**) Total energy per unit cell of Mo_6_S_3_I_6_ as a function of the lattice constant c, showing two local minima at c = 12.50 Å, and c = 13.75 Å. The letters S and L denote short and long sulfur bridge configuration, respectively.

To provide a better understanding on the atomic structure of Mo_6_S_3_I_6_, we performed density function theory (DFT) calculations and obtained two stable structures at local energy minima dependent on the elongation of Mo-S-Mo bond, which is different from the result of Tománek *et al*.^17^ Based on these two stable structures, we propose various new structural models of Mo_6_S_3_I_6_ nanowires, by changing the decorative and bridging sites of sulfur and iodine atoms linked to the two Mo_6_ octahedra in the unit cell as shown in Fig. 1. In this work, we explore the similarities and differences between two groups of isomers: short sulfur bridge conformers (S-form) and long sulfur bridge conformers (L-form). We calculated the electronic band structures of twenty-eight conformers, and predicted their detailed electronic properties. According to these calculations, we predict three structures of possible metallic conductors. Our subsequent DFT calculations also predict the probable structures of stable semiconducting configurations that have band gaps of less than 0.5 eV, and comparably unstable semi-metallic structures that have band gaps of less than 0.2 eV. To investigate atomic contributions to the electronic band structures, we performed the atom-pair analysis using the crystal orbital Hamilton population (COHP)^18,19^ method to interpret which kinds of atom-pair interactions are critical to producing the electronic band structures and intramolecular charge migration. Once the exact atomic structures of Mo_6_S_3_I_6_ are identified, we expect that Mo_6_S_3_I_6_ nanowires will be used as unique nanoscale building blocks for a wide range of potential applications. As 2D TMDCs, they are likely to be useful for fabricating efficient nanoelectronics such as sensors, optoelectronic, transistors, and photovoltaic devices^20–24^.

## Results

### Structural properties of new atomic models of Mo_6_S_3_I_6_ nanowires

The initial structure of Mo_6_S_3_I_6_ was prepared based on the previous research^10,12,14,17^. Structural parameters such as Mo-Mo bond lengths (3.24 Å) within the Mo_6_ octahedron were taken from Karthikeyan *et al*.^14^ Karthikeyan and coworkers also suggested that the bond length of Mo-S in the bridge positions (2.19 Å) is relatively shorter than those in the Mo_6_ octahedron block. Using these parameters, we constructed an initial structure and then initially optimized it with a C_3υ_ symmetry constraint by performing DFT calculations using PBE0 hybrid functional with def2-SV(P) basis set as implemented in Turbomole 7.2 program^25^. With this optimized structure, the further geometry optimizations are performed for the total 28 newly proposed atomic configurations in a hexagonal unit cell, in which the initial lattice constants are a = b = 15 Å, c = 12.5 Å and 13.75 Å. All the optimized lattice constants are determined by the volume and the ion relaxation processes for the total atomic models and reported as Table S1 in the supplementary information (SI). The two Mo_6_ octahedra in the unit cell have the same structure in C_3υ_ symmetry but are rotated by 180° from each other.

The initial structure of Mo_6_S_3_I_6_ nanowire for structural calculations is shown in Fig. 1(a): it is composed of the two Mo_6_ octahedra decorated by S- and I- atoms at the positions labeled by A*i*, A′*i*, B*j*, and B′*j*, *i* = 0–3, *j* = 1–6; (*i* = 0 refers to no sulfur atoms but three iodine atoms in the bridging plane). To begin with, the sulfur atoms in the bridging plane linked to the two Mo_6_ octahedra are placed at both P3 and P6 layers, varying *i* from 0 to 3. In this step, the maximum number of sulfur atoms can be no more than three in P3 or P6 layer but the positions of sulfur atoms can be different from P3 and P6 layers. The rest of sulfur atoms and the remaining twelve iodine atoms are assigned to the sites determined by the periodicity of nanowires and molecular symmetry kept in their stoichiometry of Mo_6_S_3_I_6_ composition. Consequently, the total of twenty-eight possible atomic models are studied in this work.

Mo_6_S_3_I_6_ nanowires have large inter-chain separations with van der Waals (weak) interactions between the chains, and the nanowires are elastic in the direction along the chains^10^. We calculated the total energy of our targeted nanowire as a function of the lattice constant c and the results are presented in Fig. 1(b). The initial structure is uniformly elongated along the uniaxial axis and the two structural energy minima were found at lattice constants c = 12.50 Å, and 13.75 Å due to bi-stability of the S_3_ linkages^17^. Though the two structural minima are very close in energy with the energy difference of only 0.03 eV/unit cell, the conformer with lattice constant c = 12.5 Å, is more stable and denoted as S (short form) and the other conformer is denoted L (long form). Accordingly, we hypothesized that the atomic configuration with which the S- and I- atom have in a unit cell would be important in determining not only the total energy but also the electronic structure of the nanowires.

**Figure 2:**
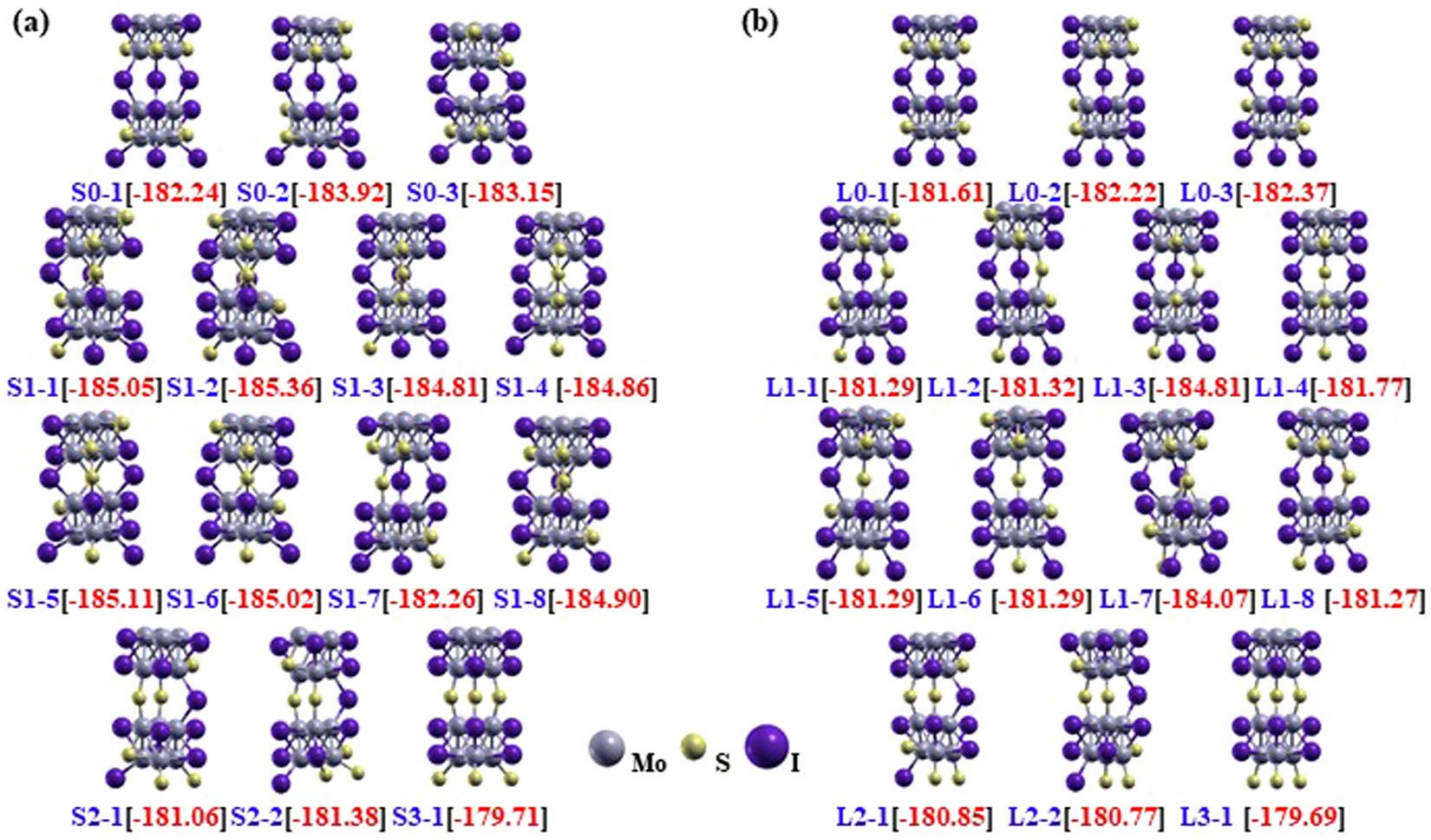
Ball and stick models of the optimized structures of various atomic Mo_6_S_3_I_6_ nanowires identified in Table 1 with their total energies (eV/unit cell) indicated in square brackets for (**a**) S-form conformers (c = 12.5 Å), and (**b**) L-form conformers (c = 13.75 Å) of Mo_6_S_3_I_6_ nanowires.

Table 1 presents a summary of the possible atomic model configurations labeled by the following convention: The first letter ‘S’ or ‘L’ represent short and long sulfur bridge conformers, respectively, of the Mo_6_S_3_I_6_ nanowires, and is followed by the number of sulfur atoms in the bridging plane. The additional number following the hyphen labels each of the possible conformers for that number of sulfur atoms in the bridging plane; zero, one, two, and three sulfur atoms in the bridging plane correspond to a total of three, eight, two, and one possible conformers, respectively. Finally, the optimized structure of these 28 atomic models are determined (Fig. 2).

**Table 1:** Atomic arrangement for various models of either short (S) or long (L) sulfur bridge configurations of Mo_6_S_3_I_6_ nanowires.

^#^Of Sulfurs in Bridging Plane	0			1								2		3
Models	S (L) 0–1	S (L) 0–2	S (L) 0–3	S (L) 1–1	S (L) 1–2	S (L) 1–3	S (L) 1–4	S (L) 1–5	S (L) 1–6	S (L) 1–7	S (L) 1–8	S (L) 2–1	S (L) 2–2	S (L) 3–1
A1	I	I	I	S	S	I	I	I	I	I	S	I	S	S
A2	I	I	I	I	I	S	S	S	I	I	I	S	S	S
A3	I	I	I	I	I	I	I	I	I	S	I	S	I	S
A′1	I	I	I	I	I	I	I	I	I	S	I	S	I	S
A′2	I	I	I	I	I	I	S	S	I	I	I	S	S	S
A′3	I	I	I	S	S	S	I	I	I	I	S	I	S	S
B1	I	I	I	I	I	S	S	I	I	I	I	I	I	I
B2	I	S	S	S	I	I	I	S	I	I	I	I	I	I
B3	I	I	I	I	S	I	I	I	I	I	I	I	I	I
B4	S	S	I	I	I	I	I	I	I	I	I	S	S	I
B5	S	I	S	I	I	I	I	I	I	S	S	I	I	I
B6	S	S	S	S	S	S	S	S	I	S	S	I	I	I
B′1	I	S	I	I	I	S	S	S	I	I	I	I	I	I
B′2	I	I	I	I	S	I	I	I	I	I	I	I	I	I
B′3	I	S	S	S	I	I	I	I	I	I	I	I	I	I
B′4	S	I	I	I	I	I	I	I	I	S	S	I	I	I
B′5	S	I	S	S	S	I	S	S	I	S	S	I	I	I
B′6	S	S	S	S	I	S	I	I	I	I	I	S	S	I

A*i*, A′*i*, B*j*, B′*j* represent the sites of sulfur or iodine atoms decorating the Mo_6_ octahedron. The sulfur atoms in the bridging plane are assigned at A*i* and A′*i* and then the remaining S and I atoms are distributed in different permutations.

The energies of S-form and L-form conformers are presented within a precision of 10^−2^ eV/unit cell for a given atomic composition. The short form conformers with only one sulfur atom in the S_3_ bridging plane (S1-*k*, *k* = 1–8) are more stable (by about 5 eV/unit cell) than the short form conformer with three sulfur atoms in the bridging plane (S3–1). We found that the S3–1 conformer, which we used as the initial structure, is the highest in energy of all the proposed structures and is therefore the least stable. It is noteworthy that the energies of S0-*k* series are lower than those of the S3–1 conformers, so the conformers with I_3_ linkages are more stable than the ones with S_3_ linkages, which is different from the previous research^17^.

**Figure 3:**
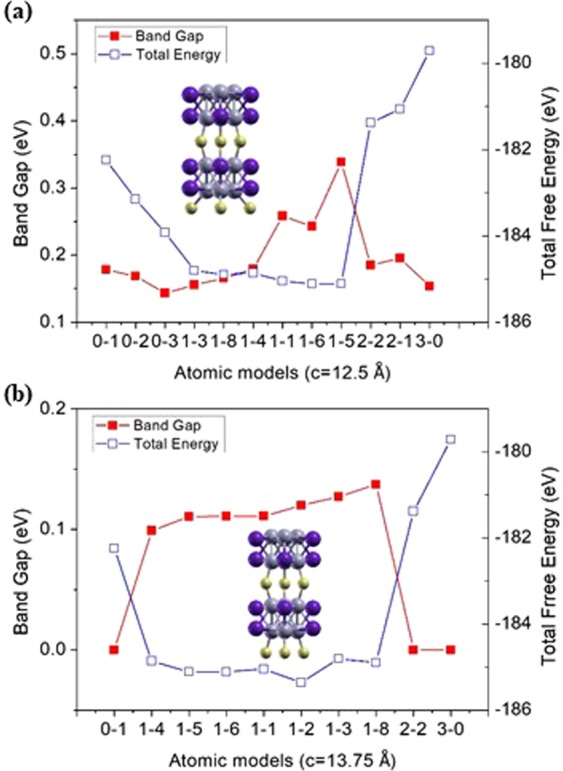
Electronic band gap energy and total energy per unit cell of various atomic models for (**a**) S-form conformers (c = 12.5 Å), and (**b**) L-form conformers (c = 13.75 Å) of Mo_6_S_3_I_6_ nanowires. The red line denotes the band gap energy, and the blue line denotes the total energy of Mo_6_S_3_I_6_ nanowire.

The characteristic feature of the optimized structures for eight conformers of the calculated S1-*k* (*k* = 1–8) series is that the conformers are distorted during the ionic relaxation due to the displacement of the sulfur atom in the bridging plane towards the center of the bridge. This happens because the bridge tries to make the possible connection through the Mo-S bond, which is extended from 2.32 Å to 2.54 Å. In addition, the angle of Mo-S-Mo at the trigonal planar in the S_3_ linkage plane that is perpendicular to the z-axis is found to change from 60 to 119.71 degrees. The unstable nanowire structures turned out to be better conductors through the ionic relaxation. Similarly, L-form structures with corresponding configurations have the same tendencies as the S-form structures regarding the displacement of their structures on a small scale. However, there is quite a remarkable distortion in the L1–7 conformer to make a strong overlap between Mo-S atoms. Like L1–7 conformers, a few of S-form and L-form conformers are hard to be bound due to their deviation in the linear correlation so that they were excluded in Fig. 3.

The total energies per unit cell of various atomic models of Mo_6_S_3_I_6_ nanowires are plotted as a function of their calculated electronic band gap energies E (k) in Fig. 3. It can be seen that the total energy is inversely related to the electronic band gap energy. This implies that the structural stability and the electronic band gap of the nanowires are inversely correlated. This relationship is due to not only van der Waals interactions between the bridge chains but also polar covalent bonds through the hybridization between the valence orbitals^14^.

### Electronic properties of new atomic models of Mo_6_S_3_I_6_ nanowire

Electron transport through the bridge chains in a Mo_6_S_3_I_6_ nanowire is known to be important for potential applications in molecular electronics^10–14,17^. To understand the effect of compositional variation on electron transport, the electronic structures and corresponding properties of the newly proposed atomic models of Mo_6_S_3_I_6_ nanowire were investigated.

**Figure 4:**
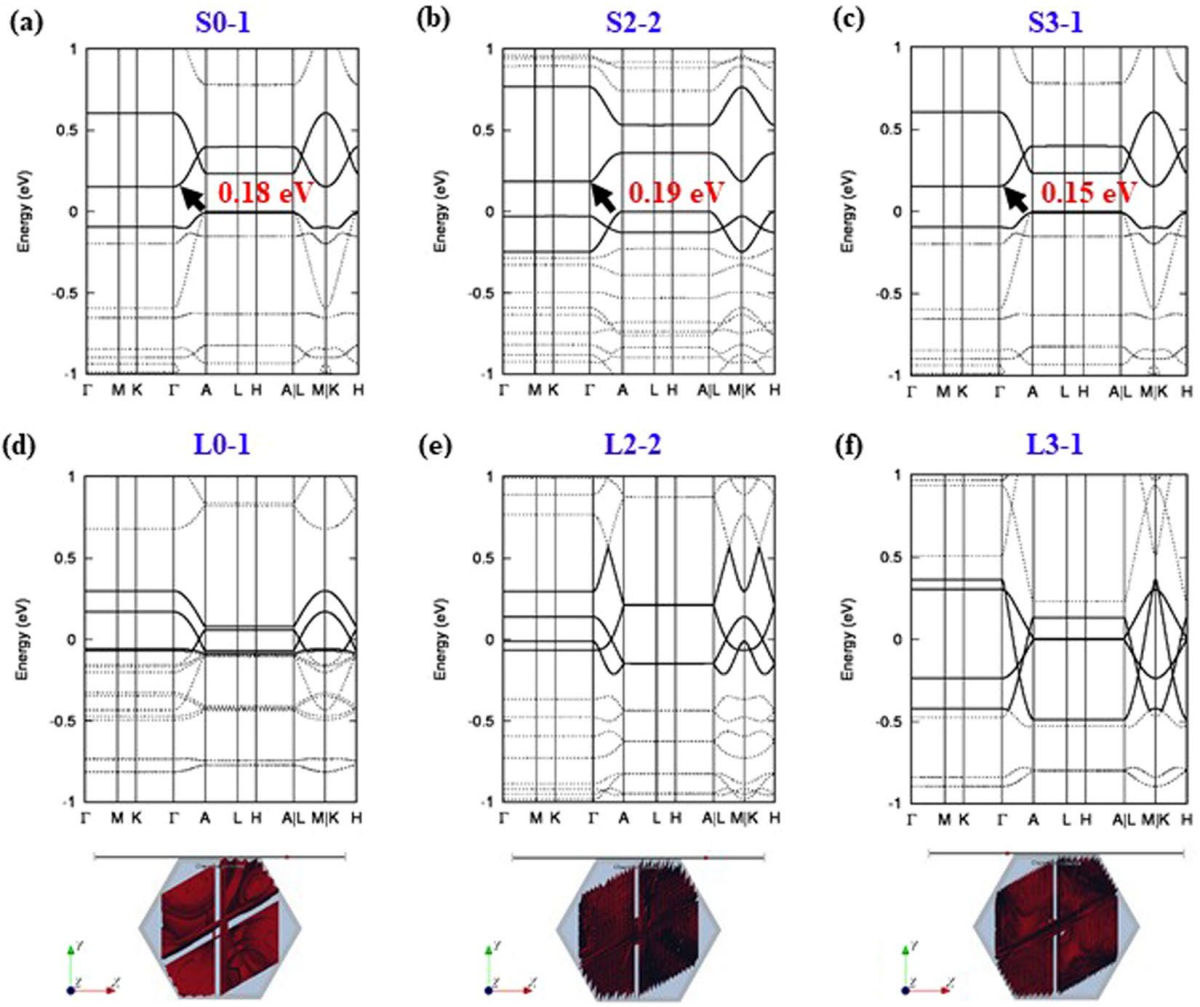
Electronic band structure of (**a**) S0–1, (**b**) S2–2, (**c**) S3–1, (**d**) L0–1, (**e**) L2–2, and (**f**) L3–1 conformers of a Mo_6_S_3_I_6_ nanowire. The conduction and valence bands are emphasized by bold lines. While (**a**–**c**) are semiconductors, (**d**–**f**) are all metallic conductors, whose Fermi surfaces at each Fermi energy are shown below their electronic band structures with 3 times denser k-meshes using the interpolation method implemented in BolzTrap2^46^ program. The Fermi surface for L0–1 is less dense than the ones for L2–2 and L3–1 conformers. The characteristic feature is to have a bridging connection in the middle of Fermi surfaces for those three metallic conformers shown in (**d**–**f**).

The electronic band structure along with reciprocal symmetry lines of S-form conformers and those of the corresponding L-form conformers are shown in Fig. 4. It is apparent that S0–1, S2–2, S3–1 conformers are narrow-gap semiconductors having band gap energies of 0.18 eV, 0.19 eV, and 0.15 eV, respectively. Of the conformers studied, the band gap of the S1–5 conformer is the largest at 0.34 eV, while that of the S3–1 conformer is the smallest of the S-form conformers (see Figs S8 and S14 in SI). The band gap energy is larger for more stable structures. It is more obvious that structural stability is inversely correlated with the electronic band gap in the case of L-form conformers, as presented in Fig. 3. Since the most of band gap energies in L-form conformers are all less than 0.2 eV, the L-form conformers can be regarded as narrow-gap semiconductors. The band structures of other atomic models in S-form and L-form conformers are presented in SI (see Figs S1–S28 in SI).

**Figure 5:**
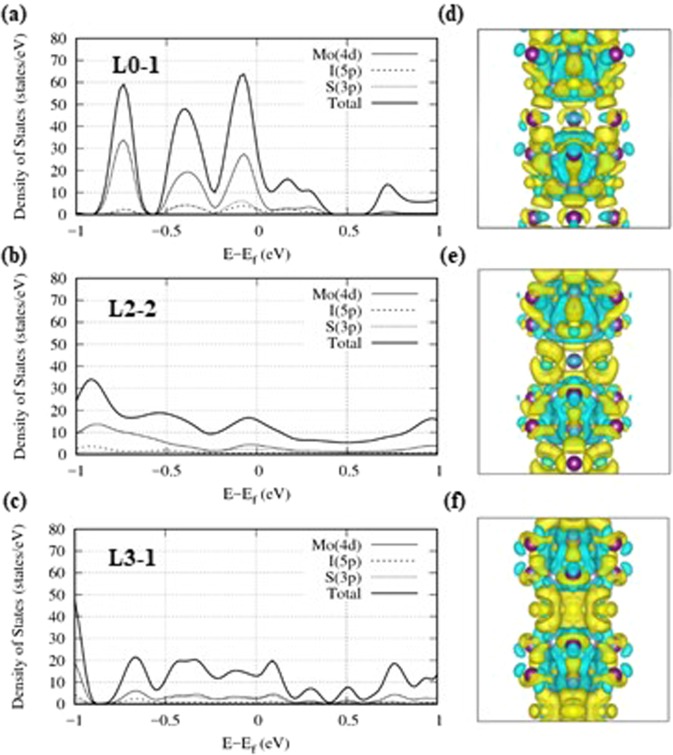
Projected density of states (pDOS) for three metallic conformers, (**a**) L0–1, (**b**) L2–2, and (**c**) L3–1 of a Mo_6_S_3_I_6_ nanowire. The contributions of Mo, S, and I atoms are plotted individually for comparison. The main contributions to the conduction band are from Mo-4d, S-3p, and I-5p orbitals. (**d**–**f**) Valence charge density differences (VCDDs) in the three corresponding metallic conformers at c = 13.75 Å of Mo_6_S_3_I_6_ nanowire. The yellow and cyan denote regions of charge depletion and excess, respectively, with respect to the superposition of isolated atoms.

Figure 5(a–c) display the projected density of states (pDOS) of a Mo_6_S_3_I_6_ nanowire in the energy range of −0.1 eV ≤ E-E_F_ ≤ 0.1 eV. The Fermi energy (E_F_) is close to the top of the valence band and crosses the hybridized bands belonging to molybdenum, sulfur, and iodine. Small dispersion of the sub-bands together with the finite DOS at E_F_ is responsible for the semi-metallic and metallic transport properties of these nanowires^14^. Since the DOS at E_F_ is nonzero, we could expect that L0–1, L2–2, and L3–1 conformers are conductors that could be varied by their composition and elongation of the nanowire. It is supposed that a periodic distortion of the bond lengths somehow influences the behavior of the electrons in these systems because of a Peierls instability^26,27^. We find that structural instability causes the fluctuations of charge density waves^28,29^. As the electron density at E_F_ increases, the number of band crossings at the Fermi level increases. Several interpenetrating sub-bands, three in particular, cross the Fermi level through the reciprocal symmetry line of Γ-Α as shown in Fig. 4(d–f).

Close to the Fermi level, the hybridization of Mo-4d, S-3p, and I-5p contributes to forming quasi-1D sheets^27,28^ or Fermi surfaces. It is obvious that the electron density close to the Fermi level of the L3–1 conformer is the highest, and is more equally distributed than the L0–1 conformer as shown in Fig. 5(a–c). It causes that the Fermi surface for L0–1 is less dense than the ones for L2–2 and L3–1. It is noteworthy that the DOS at E_F_ is important for Fermi surfaces because there are sub-bands penetrating through the Fermi level. The pDOS of the calculated atomic models are presented in SI (see Figs S1–S28 in SI).

So far, the effect of the compositional variation on electron transport, electronic structures of our newly proposed Mo_6_S_3_I_6_ nanowire configurations have been discussed. Moreover, the impact of the Mo-S bridge chains in a Mo_6_S_3_I_6_ nanowire on charge density must also be understood for future applications.

The valence charge density differences (VCDDs) are calculated as a difference between the total charge density of the system and the superposition of the valence charge densities of neutral atoms^30^. The valence charge density differences in three metallic conformers are shown in Fig. 5(d–f). The yellow presents an accumulation of negative charges, whereas cyan denotes a depletion of charges as compared to neutral atoms. It is clearly seen that the excess valence charges between Mo-S bridge chains increase from the L0–1 to the L3–1 conformers. As the excess charge densities between molybdenum and sulfur atoms in the bridging plane increase, the nanowires are expected to become better conductors due to their electron delocalization. Since the conduction band charge of the Mo-4d orbitals is mostly rich enough to be transferred to the sulfur atoms through the polar covalent bond of Mo-S^11^, we guess that the Mo-S interaction plays a key role in charge transport.

Previous studies with partial DOS have elucidated the electronic structures of these materials, however, the nature of states at the Fermi level have not been characterized by chemical-bonding analysis^31,32^. Using crystal orbital Hamilton population (COHP) curves implemented in the Local-Orbital Basis Suite Towards Electronic-Structure Reconstruction (LOBSTER) package^31,33^, we obtained the information about bonding and antibonding contributions of our targeted Mo_6_S_3_I_6_ nanowires by re-extracting the atom-resolved information from delocalized plane-wave basis sets.

**Figure 6:**
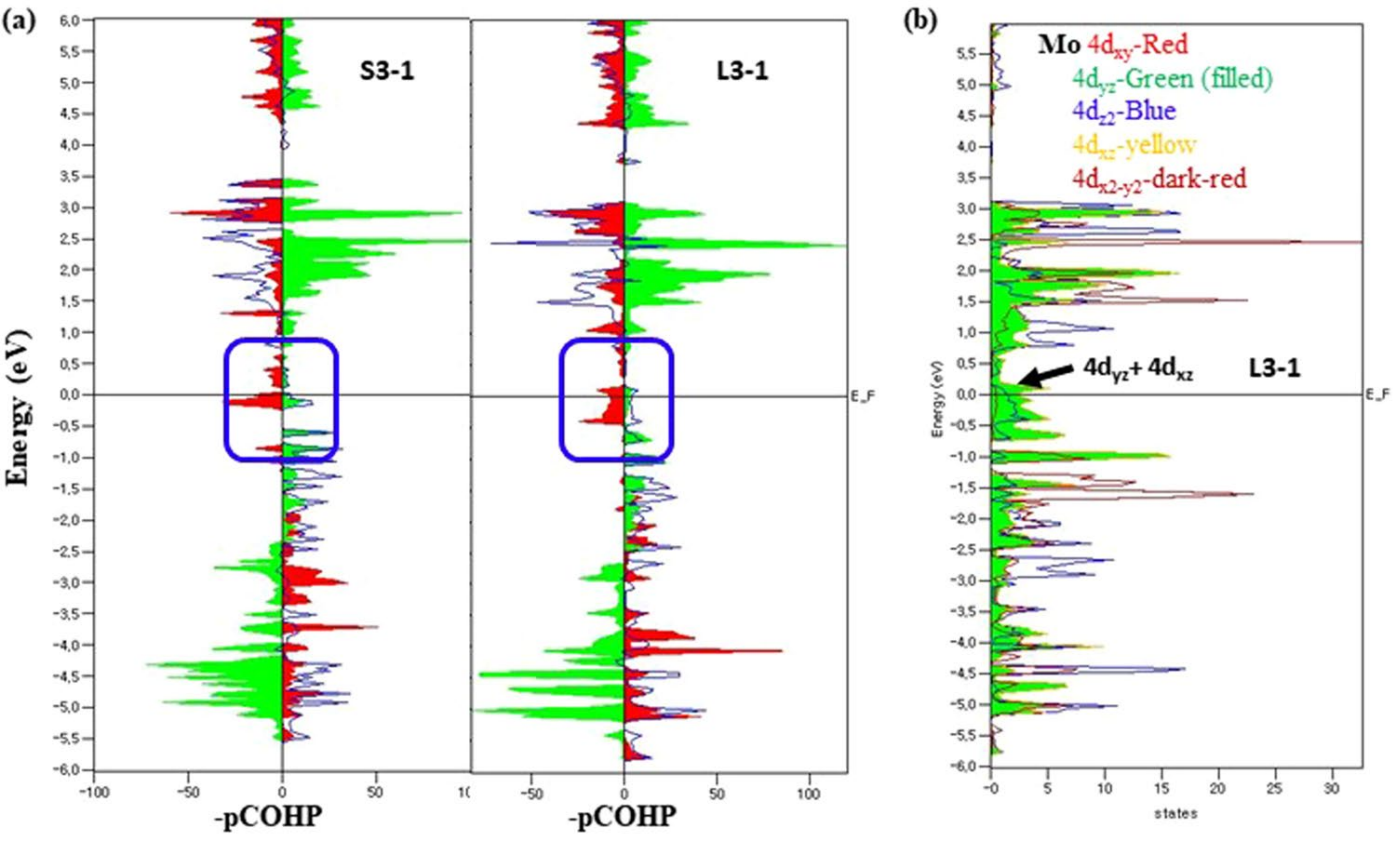
(**a**) The atom-projected crystal orbital Hamilton population (pCOHP) of S3–1 vs L3–1 conformers of a Mo_6_S_3_I_6_ nanowire. The bonding orbital-pair interactions are presented on the right side of the panel, while the antibonding interactions are on the left side. The pCOHP of Mo-4d and S-3p are indicated as blue lines and red filled shadows, respectively, whereas the contributions from I-5p states are indicated as filled green shadows with sign flips for better comparison. (**b**) The orbital-pDOS of Mo-4d for the L3-1 metallic conformer. The red, filled green, blue, yellow, and dark-red denote 4d_xy_, 4d_yz_, 4d_z2_, 4d_xz_, and 4_dx2−y2_ orbital contributions, respectively. The energy axis is shown relative to the Fermi level.

The atom-pCOHP of the S3–1 and L3–1 conformers of a Mo_6_S_3_I_6_ nanowire are shown in Fig. 6(a). The diagram of COHP reveals a stabilizing or destabilizing energy criterion that converts the DOS into both negative for bonding and positive for antibonding values, in contrast to the conventional DOS, which yields the number of electrons in the system^32^. As shown in Fig. 6(a), the orbital energy is plotted as a function of the negative value of projected crystal orbital Hamilton population (–pCOHP) for convenience. Molybdenum shows the bonding character, but the sulfur and iodine show the antibonding character both in the valence band and near the Fermi level. Particularly, a much larger antibonding character of the sulfur atoms can be found near the Fermi level compared to that of iodine atoms; the antibonding character of sulfur atoms contributes to the metallic nature of a Mo_6_S_3_I_6_ nanowire by destabilizing the band structure energy. In addition, it is obvious that the dominant contributions are from 4d_yz_ and 4d_xz_ orbitals of Mo as shown in Fig. 6(b). We found that the sulfur contributions are mainly from 3p_x_ and 3p_y_ orbitals, and from 5p_x_ and 5p_y_ orbitals for iodine (see Fig. S29 in SI).

COHP partitions the band-structure energy into orbital-pair interactions between a pair of adjacent atoms. A COHP diagram indicates bonding and antibonding contributions to the band-structure energy in terms of DOS that usually shows where electrons are in a system. Whereas COHP shows the contribution of a specific bond to the band energy, the integrated COHP (ICOHP) gives a hint towards the bond strength in energy unit (eV).

**Figure 7:**
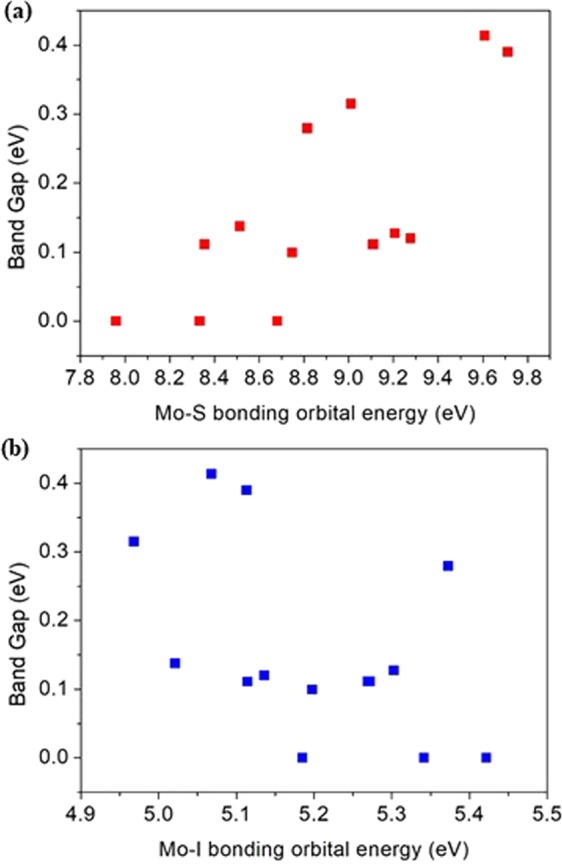
The relationship between integrated crystal orbital Hamilton populations (ICOHP) and the band gap energy of the L3–1 conformer for (**a**) Mo-S interactions, and (**b**) Mo-I interactions.

Figure 7 shows the relationship between ICOHP and band gap energy for the L3–0 conformer of a Mo_6_S_3_I_6_ nanowire based on the type of atom-pair interactions. For the Mo-S atom-pair bonding interaction, it is apparent that an increase in the bonding orbital energy stabilizes the band structure energy, which leads to an increase of the band gap energy. On the other hand, this is not the case for the Mo-I atom-pair interaction. Our work shows that the Mo-S bonding interaction is mainly responsible for not only the structural stability but also the electronic properties of Mo_6_S_3_I_6_ nanowire. The pCOHP of atom-pair interaction (Mo-Mo, Mo-S, and Mo-I) of newly proposed atomic models are presented in SI. (see Figs S30–S57 in SI).

## Conclusion

We investigated the effect of structural disorder on Mo_6_S_3_I_6_ nanowires by compositional modeling for two local energy minima structures identified by the elongation of the bridge chains. Based on the two stable structures, we performed DFT calculations to explore the impact of sulfur or iodine atom locations on the electronic properties of the newly proposed atomic models. In this paper, we report the structural properties, electronic band structures, and pDOS of our newly proposed atomic models. We showed that the electronic band gap energy is inversely correlated to structural stability, and introduced Fermi surfaces for the three structures with a lattice constant of c = 13.75 Å that are possible conductors. As the delocalized valence charge density differences are increased through the Mo-S bridging chains, the electron densities at the Fermi level are also increased. This implies that the existence of sulfur atoms in the bridging plane plays an important role in the intramolecular charge transport. Our theoretical calculations using crystal orbital Hamilton populations (COHP) analysis predict that the electronic band gap energy of a Mo_6_S_3_I_6_ nanowire is quite linearly correlated with Mo-S bonding or antibonding orbital energy due to the structural stability. Since compositional variation can be used to control the Mo-S bonding interactions, isolated or bundled Mo_6_S_3_I_6_ nanowires are expected to be utilized as essential components of a wide range of applications such as optoelectronics, transistors, sensors, and photovoltaic devices^20–24,34^ in near-term.

## Methods

To investigate the structural and electronic properties of an isolated Mo_6_S_3_I_6_ nanowire, we performed DFT calculations with projected augmented wave method^35,36^ and a plane-wave basis set as implemented in the Vienna *Ab initio* Simulation Package (VASP)^37–40^. The electron-electron correlation energy was corrected by the Perdew, Burke, and Enzerhoff (PBE) generalized gradient approximation (GGA)^41,42^. Ionic and electronic relaxations were carried out using an iterative conjugate gradient minimization method. The energy cut-off was chosen to be 500 eV, and Gaussian smearing for geometry optimization and Fermi smearing for band structure calculations with Blöchl correction were used with a 0.05 eV smearing width. To describe the infinite isolated nanowires with a different compositional arrangement, we placed nanowires in a large hexagonal unit cell with 15 Å vacuum space in the *x*- and *y*-directions to limit inter-wire interactions. All geometries were optimized without any symmetry constraints. The Brillouin zone was sampled by 1 × 1 × 14 Γ-centered automatic *k*-meshes to converge the ionic relaxation calculation and 100 *k*-points along the reciprocal symmetry lines to obtain the DOS. The Mo-4d, S-3p, and I-5p electrons are considered to be valence electrons not only for the pDOS but also for the calculation of crystal orbital Hamilton population, which is employed for the analysis of bonding and antibonding orbital energy and the interaction between specific atoms. In addition to VASP, Xcrysden^43,44^, wxDragon^45^ and LOBSTER^31,32^ were employed for visualizing the calculation results.

## Supplementary information

Structural and electronic properties of Mo<sub>6</sub>S<sub>3</sub>I<sub>6</sub> nanowires by newly proposed theoretical compositional ordering
