# Supplementary material for: Structural and electronic properties of Mo6S3I6 nanowires by newly proposed theoretical compositional ordering
Source: Sci Rep. 2019 Feb 4;9:1222. doi: 10.1038/s41598-018-37818-7 (PMC6362008; doi:10.1038/s41598-018-37818-7)
Supplement: Supplementary file 1 — Structural and electronic properties of Mo6S3I6 nanowires by newly proposed theoretical compositional ordering [file 41598_2018_37818_MOESM1_ESM.docx]

**Supplementary Information:**

**Structural and electronic properties of** **Mo_6_S_3_I_6_ nanowires by newly proposed theoretical compositional ordering**

You Kyoung Chung^1^, Weon-Gyu Lee^1^, Sudong Chae^2^, Jae-Young Choi^2,3,^* and Joonsuk Huh^1,^*^,^

^1^Department of Chemistry, Sungkyunkwan University, Suwon 16419, Korea.

^2^School of Advanced Materials Science & Engineering, Sungkyunkwan University, Suwon 16419, Republic of Korea.

^3^SKKU Advanced Institute of Nanotechnology (SAINT), Sungkyunkwan University, Suwon 16419, Republic of Korea

*Corresponding authors: [jy.choi@skku.edu](mailto:jy.choi@skku.edu), [joonsukhuh@skku.edu](mailto:joonsukhuh@skku.edu)


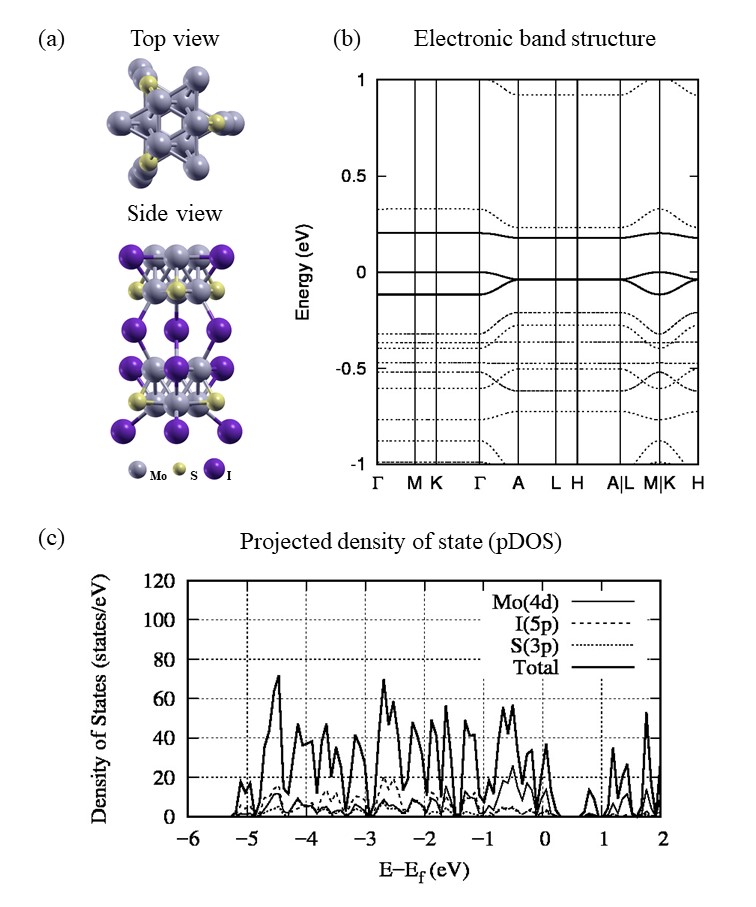


**Figure. S1** Ball and stick atomic structure (Top and Side view), (b) Electronic band structure, (c) Projected density of states (pDOS) of **S0-1** Mo_6_S_3_I_6_ nanowire


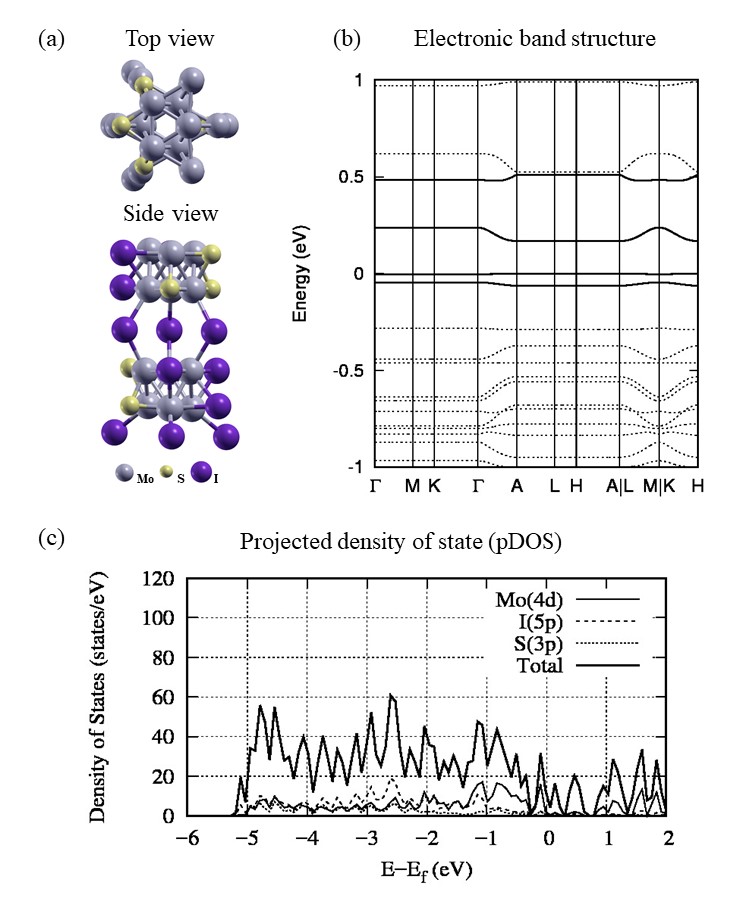


**Figure. S2** Ball and stick atomic structure (Top and Side view), (b) Electronic band structure, (c) Projected density of states (pDOS) of **S0-2** Mo_6_S_3_I_6_ nanowire


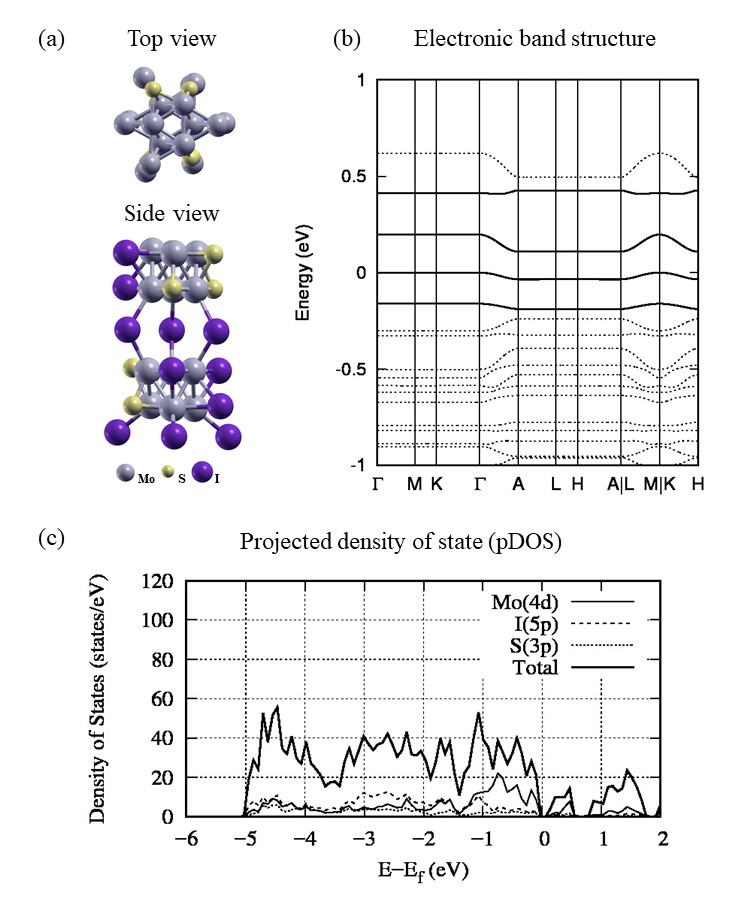


**Figure. S3** Ball and stick atomic structure (Top and Side view), (b) Electronic band structure, (c) Projected density of states (pDOS) of **S0-3** Mo_6_S_3_I_6_ nanowire


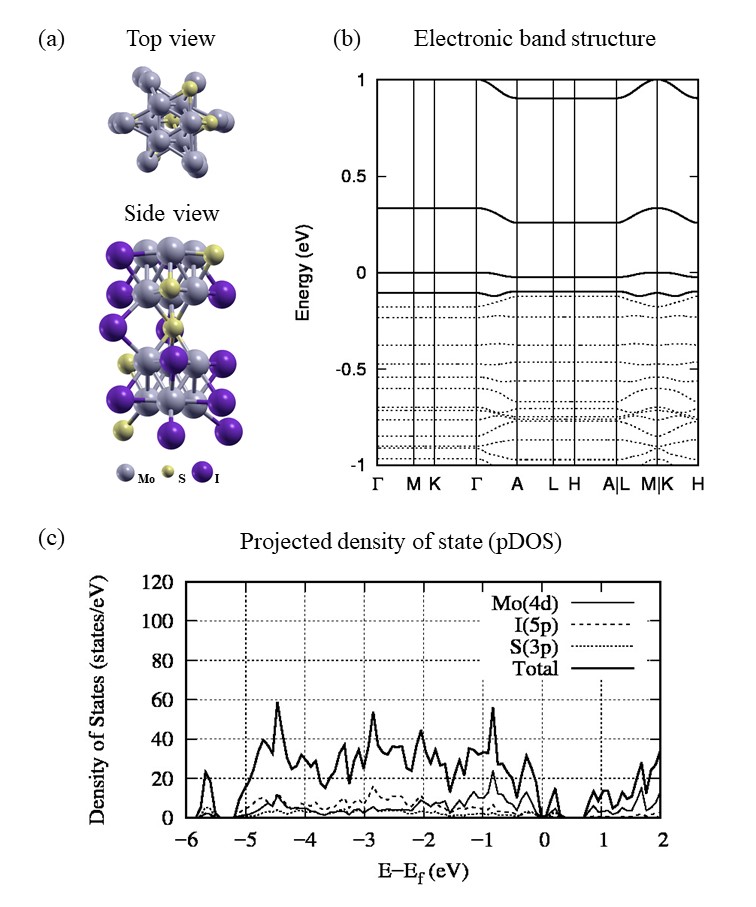


**Figure. S4** Ball and stick atomic structure (Top and Side view), (b) Electronic band structure, (c) Projected density of states (pDOS) of **S1-1** Mo_6_S_3_I_6_ nanowire


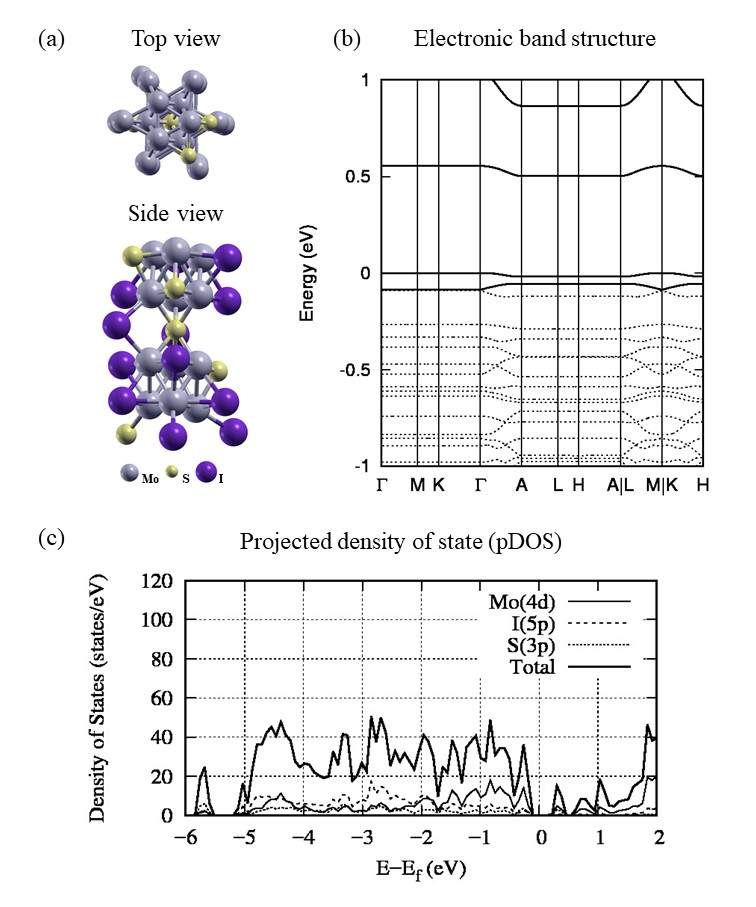


**Figure. S5** Ball and stick atomic structure (Top and Side view), (b) Electronic band structure, (c) Projected density of states (pDOS) of **S1-2** Mo_6_S_3_I_6_ nanowire


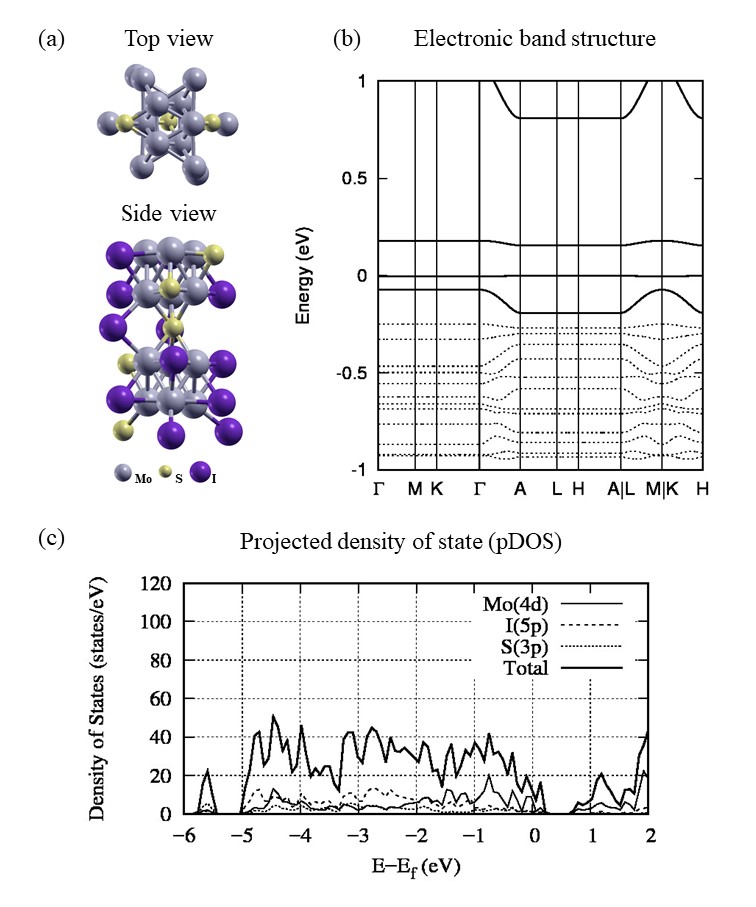


**Figure. S6** Ball and stick atomic structure (Top and Side view), (b) Electronic band structure, (c) Projected density of states (pDOS) of **S1-3** Mo_6_S_3_I_6_ nanowire


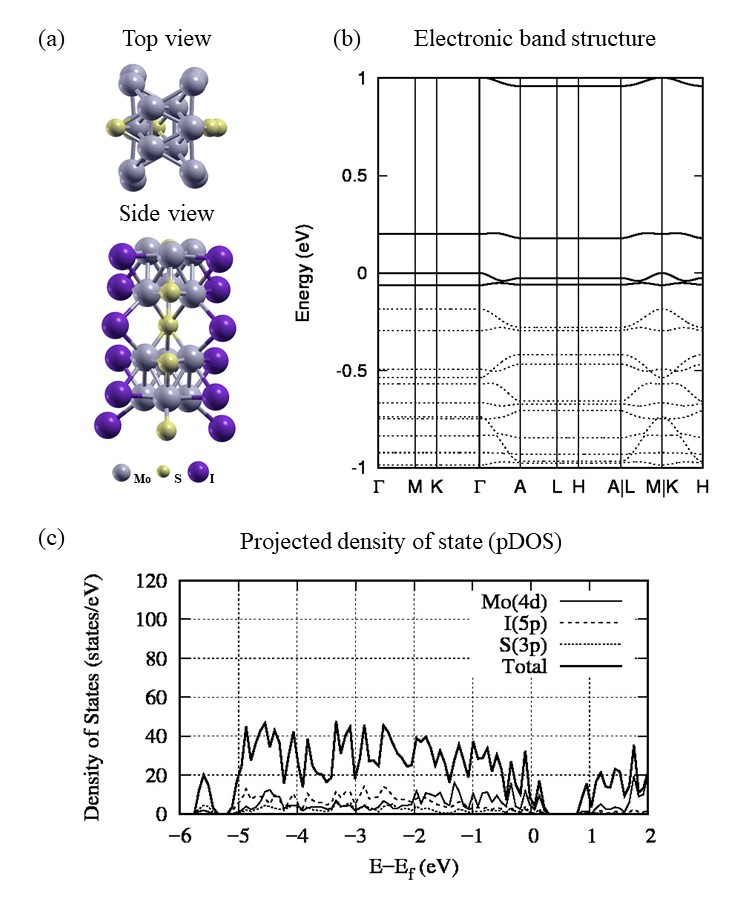


**Figure. S7** Ball and stick atomic structure (Top and Side view), (b) Electronic band structure, (c) Projected density of states (pDOS) of **S1-4** Mo_6_S_3_I_6_ nanowire


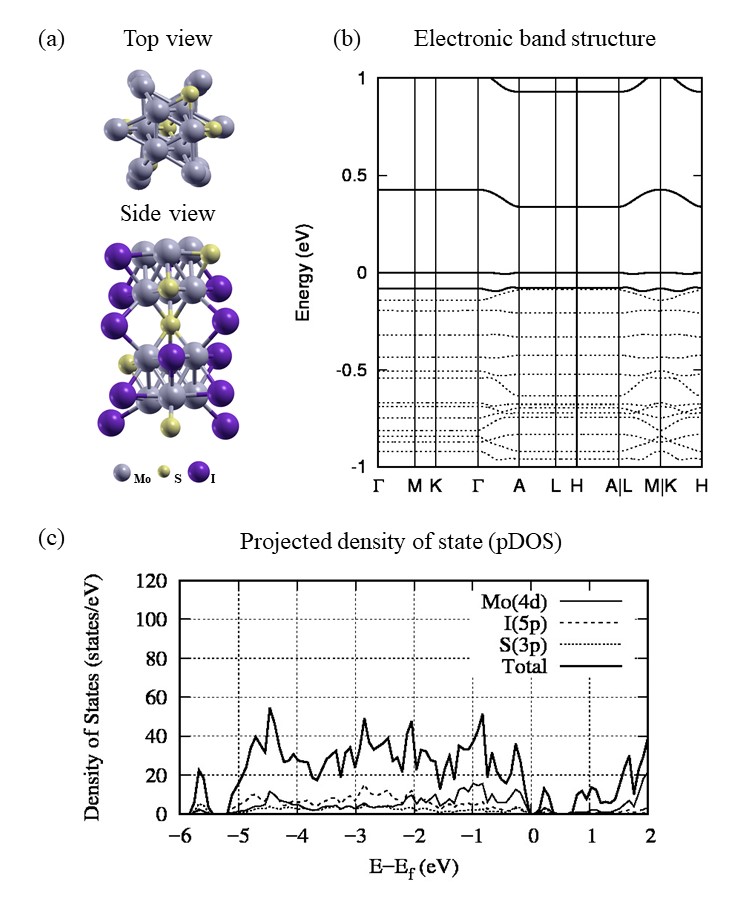


**Figure. S8** Ball and stick atomic structure (Top and Side view), (b) Electronic band structure, (c) Projected density of states (pDOS) of **S1-5** Mo_6_S_3_I_6_ nanowire


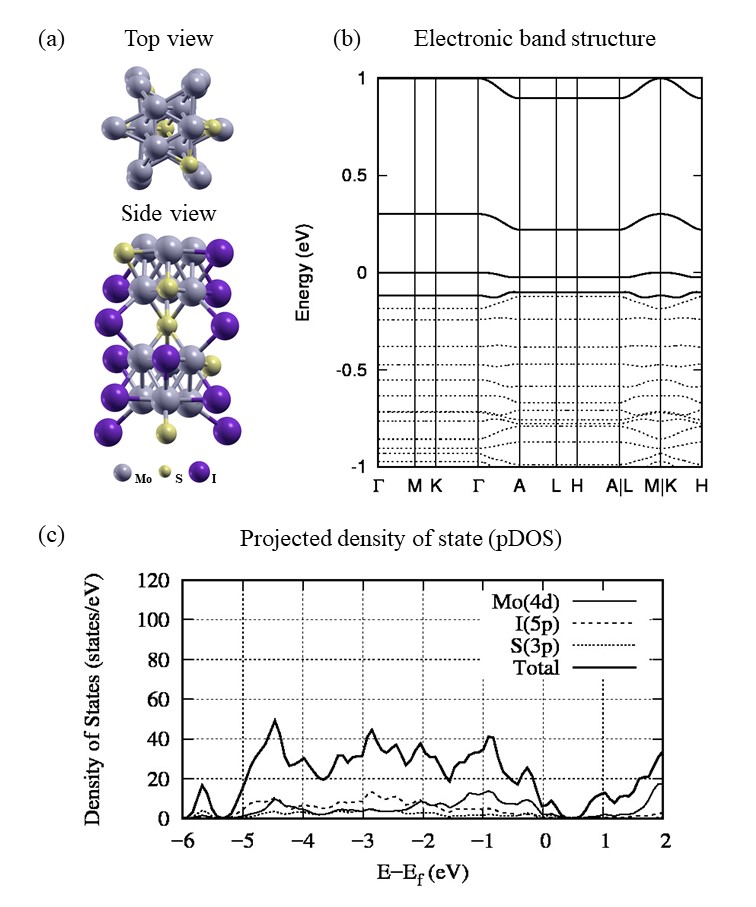


**Figure. S9** Ball and stick atomic structure (Top and Side view), (b) Electronic band structure, (c) Projected density of states (pDOS) of **S1-6** Mo_6_S_3_I_6_ nanowire


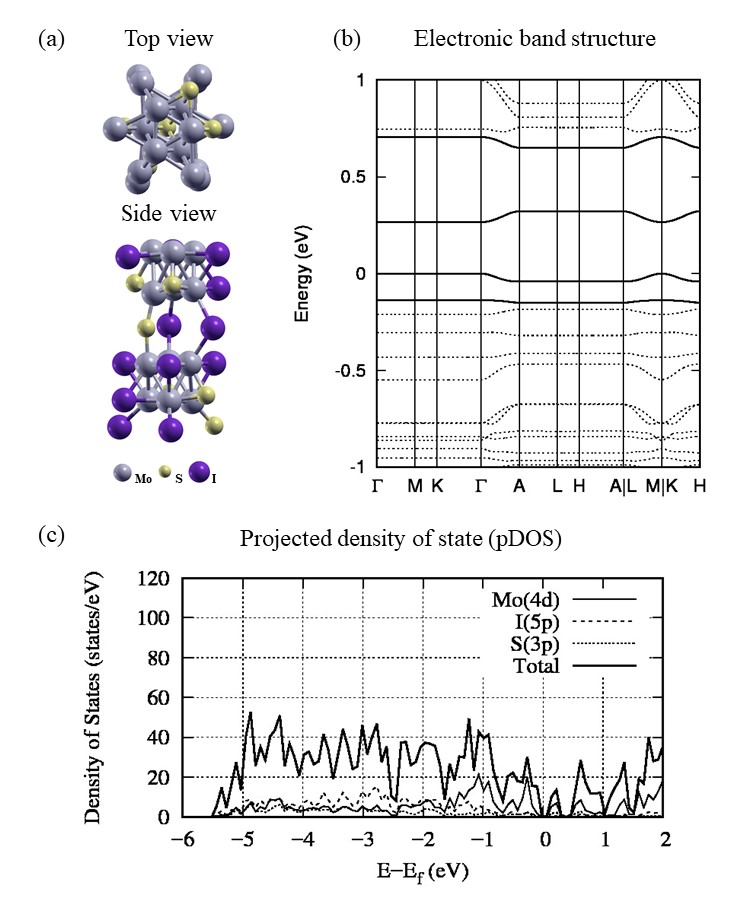


**Figure. S10** Ball and stick atomic structure (Top and Side view), (b) Electronic band structure, (c) Projected density of states (pDOS) of **S1-7** Mo_6_S_3_I_6_ nanowire


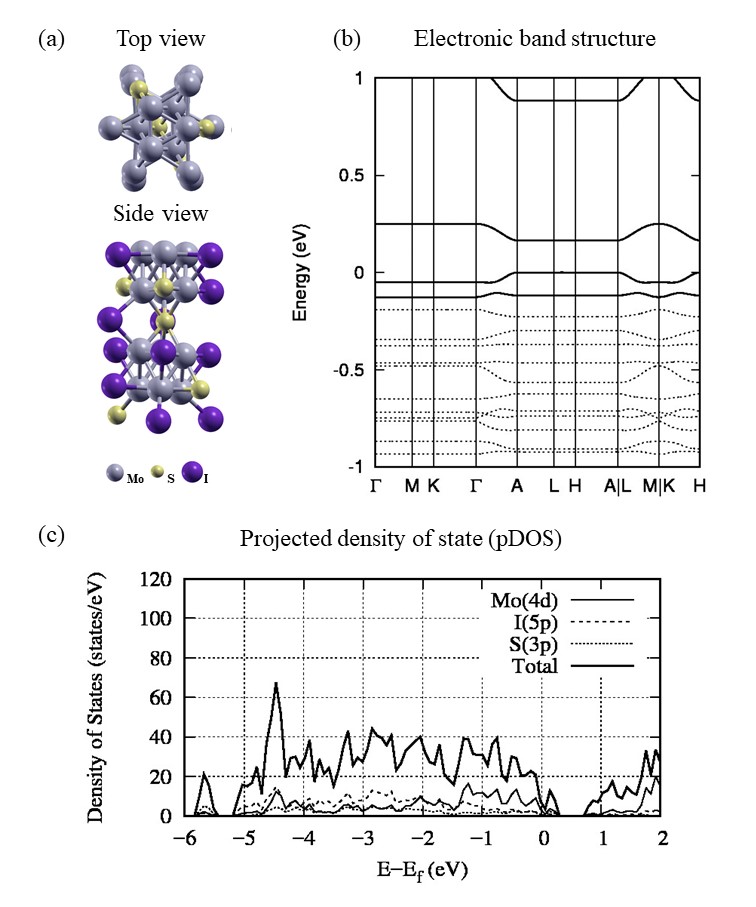


**Figure. S11** Ball and stick atomic structure (Top and Side view), (b) Electronic band structure, (c) Projected density of states (pDOS) of **S1-8** Mo_6_S_3_I_6_ nanowire


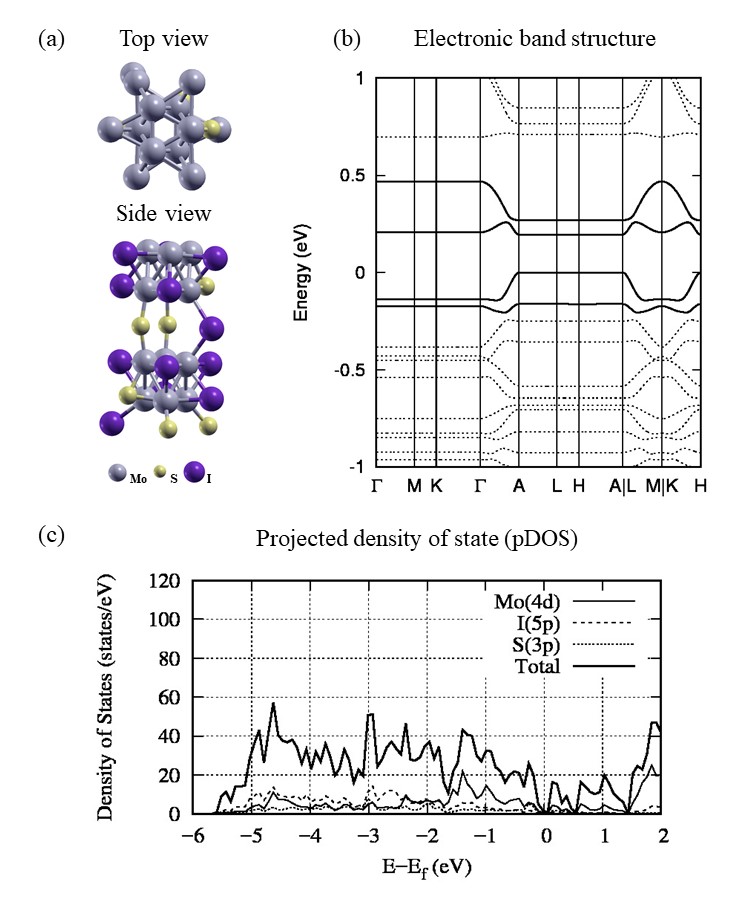


**Figure. S12** Ball and stick atomic structure (Top and Side view), (b) Electronic band structure, (c) Projected density of states (pDOS) of **S2-1** Mo_6_S_3_I_6_ nanowire


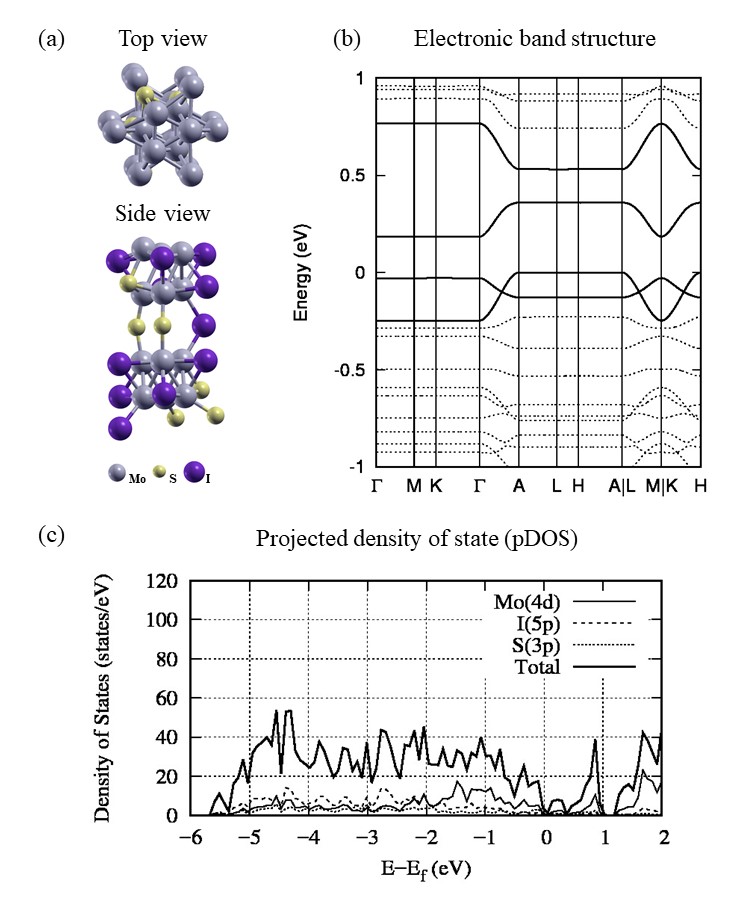


**Figure. S13** Ball and stick atomic structure (Top and Side view), (b) Electronic band structure, (c) Projected density of states (pDOS) of **S2-2** Mo_6_S_3_I_6_ nanowire


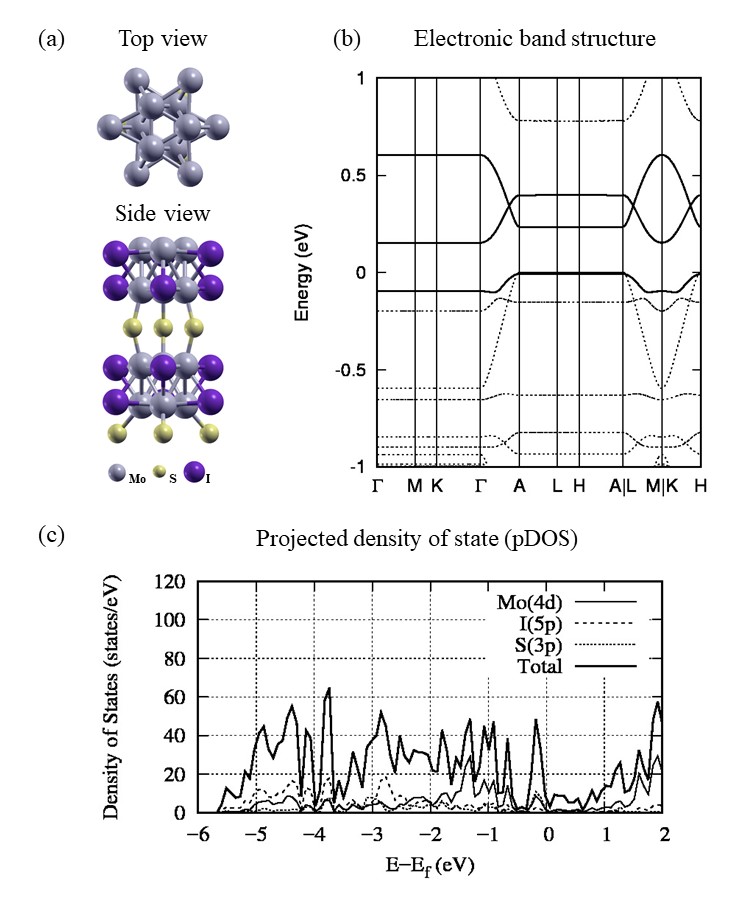


**Figure. S14** Ball and stick atomic structure (Top and Side view), (b) Electronic band structure, (c) Projected density of states (pDOS) of **S3-1** Mo_6_S_3_I_6_ nanowire


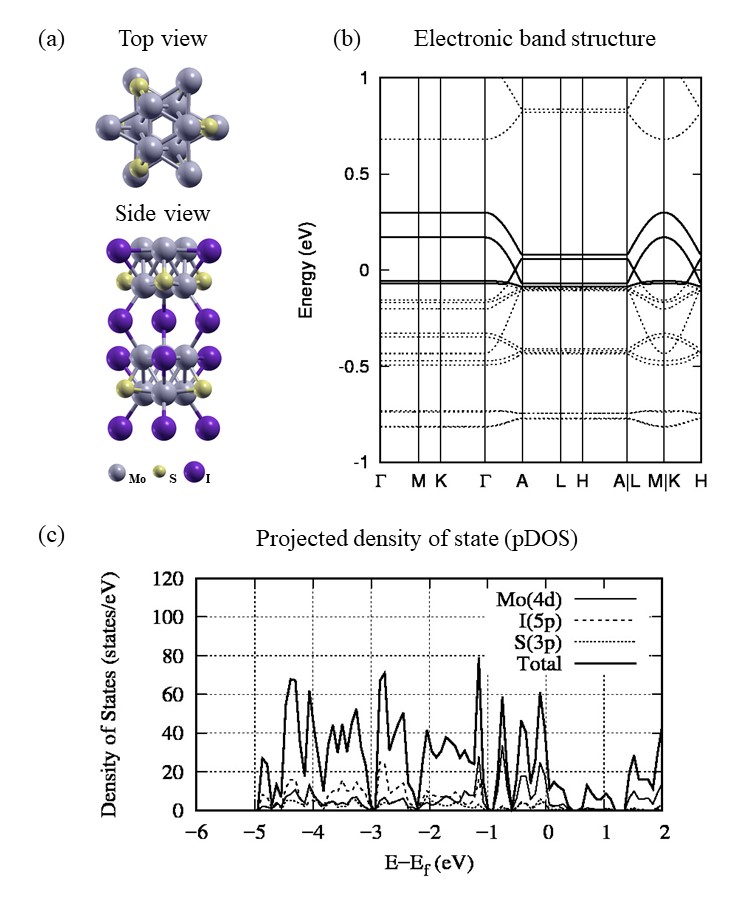


**Figure. S15** Ball and stick atomic structure (Top and Side view), (b) Electronic band structure, (c) Projected density of states (pDOS) of **L0-1** Mo_6_S_3_I_6_ nanowire


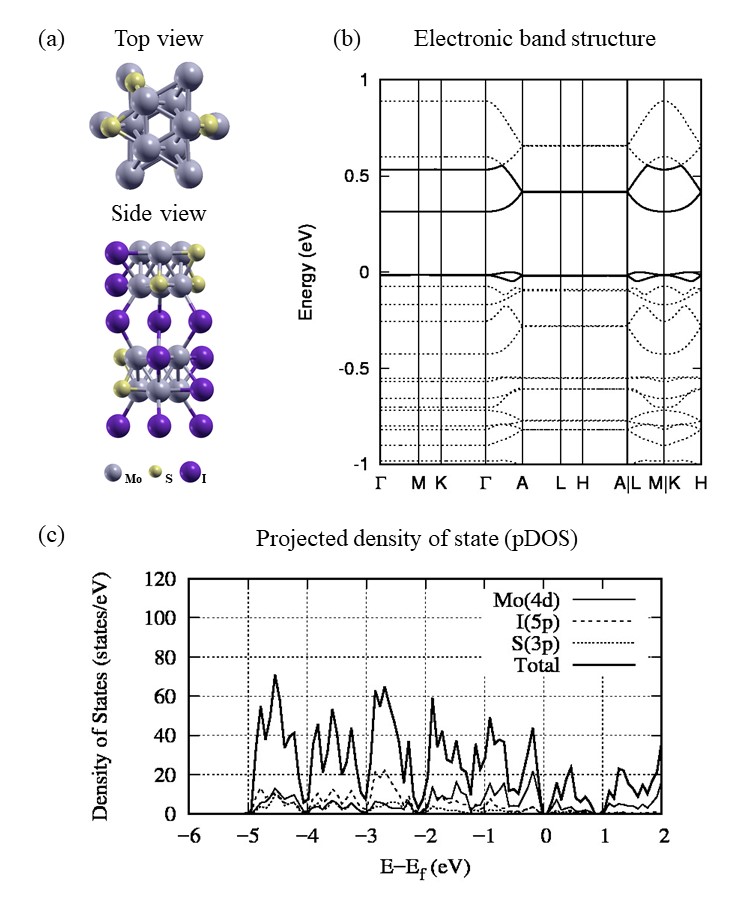


**Figure. S16** Ball and stick atomic structure (Top and Side view), (b) Electronic band structure, (c) Projected density of states (pDOS) of **L0-2** Mo_6_S_3_I_6_ nanowire


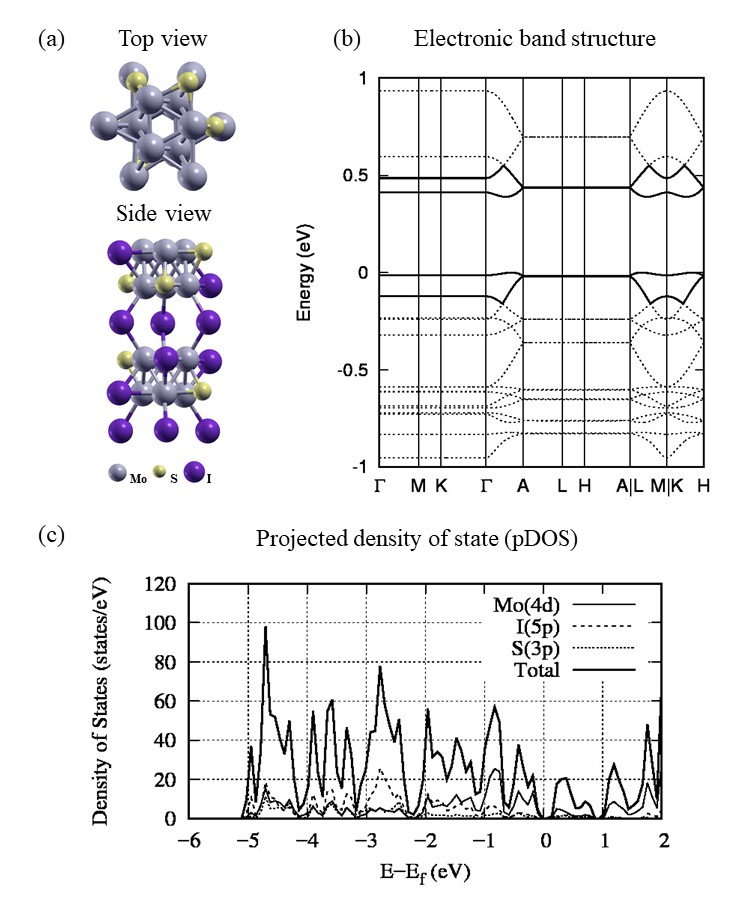


**Figure. S17** Ball and stick atomic structure (Top and Side view), (b) Electronic band structure, (c) Projected density of states (pDOS) of **L0-3** Mo_6_S_3_I_6_ nanowire


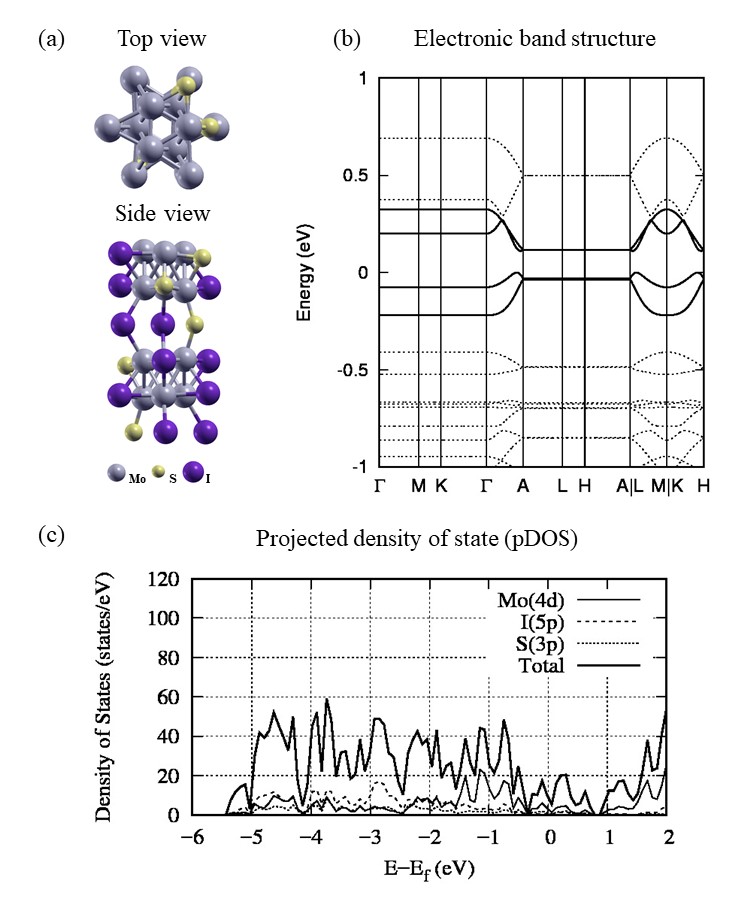


**Figure. S18** Ball and stick atomic structure (Top and Side view), (b) Electronic band structure, (c) Projected density of states (pDOS) of **L1-1** Mo_6_S_3_I_6_ nanowire


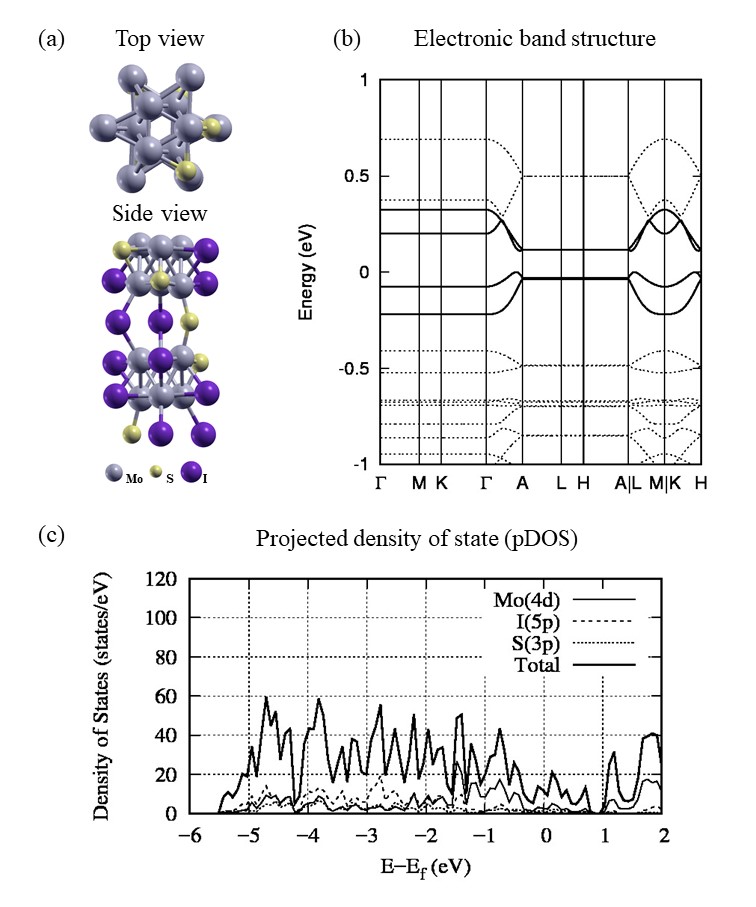


**Figure. S19** Ball and stick atomic structure (Top and Side view), (b) Electronic band structure, (c) Projected density of states (pDOS) of **L1-2** Mo_6_S_3_I_6_ nanowire


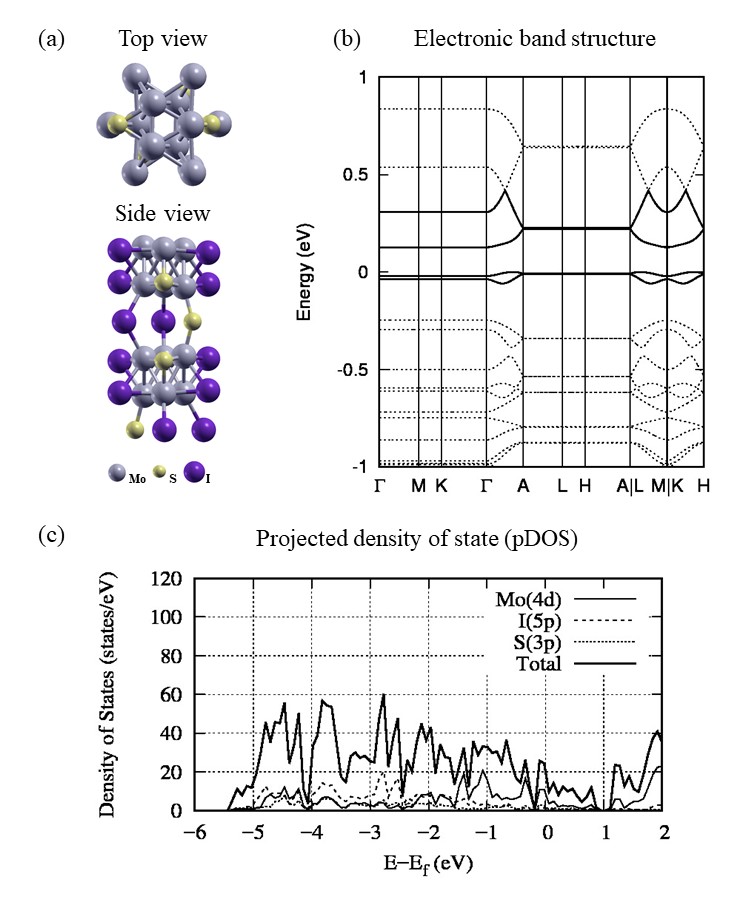


**Figure. S20** Ball and stick atomic structure (Top and Side view), (b) Electronic band structure, (c) Projected density of states (pDOS) of **L1-3** Mo_6_S_3_I_6_ nanowire


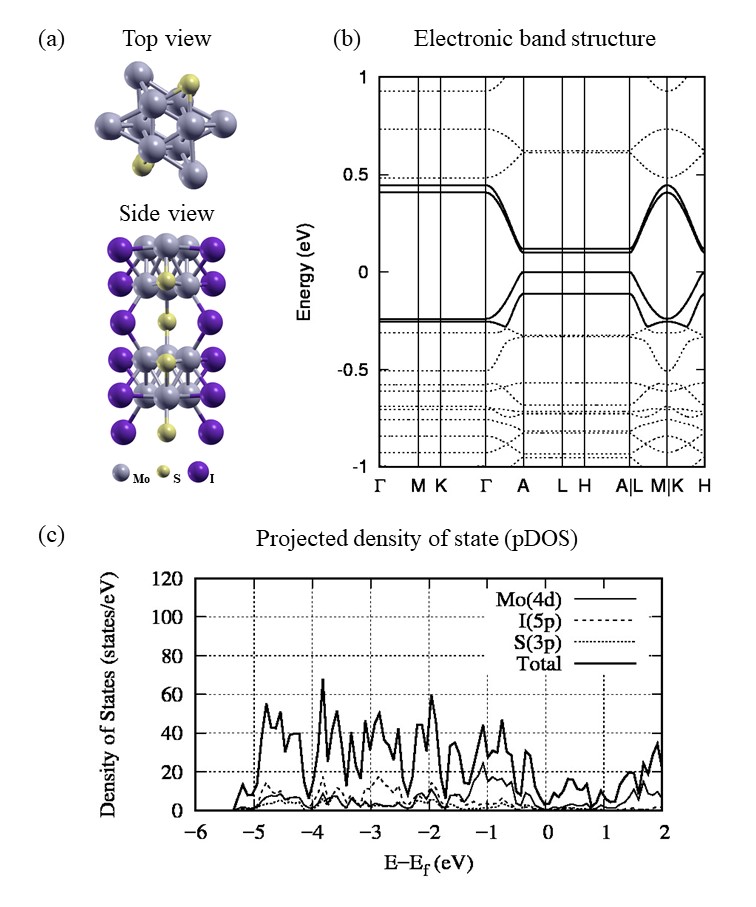


**Figure. S21** Ball and stick atomic structure (Top and Side view), (b) Electronic band structure, (c) Projected density of states (pDOS) of **L1-4** Mo_6_S_3_I_6_ nanowire


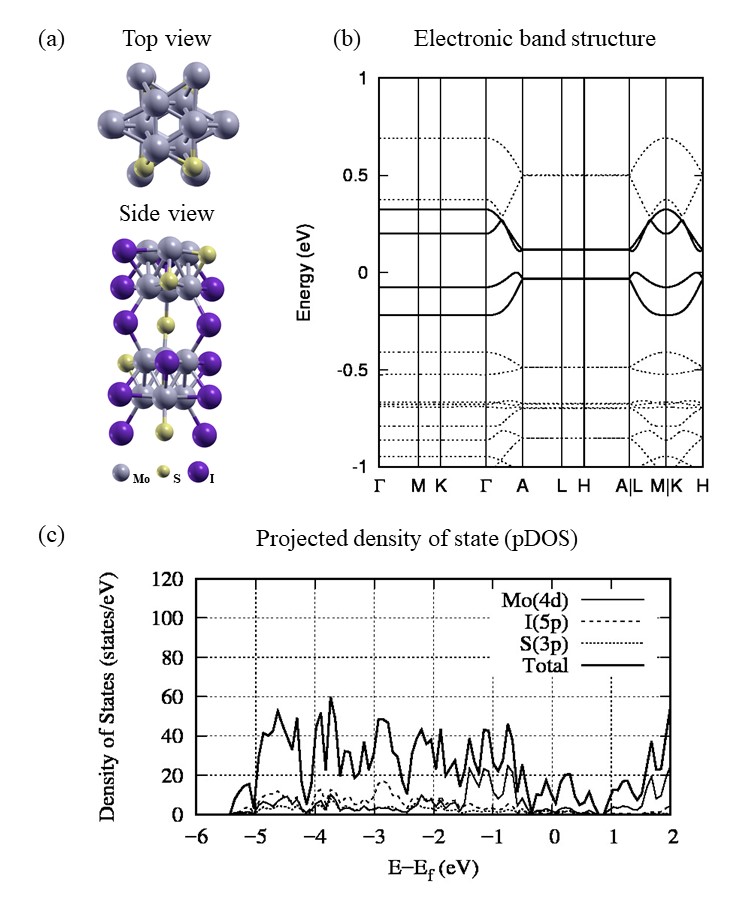


**Figure. S22** Ball and stick atomic structure (Top and Side view), (b) Electronic band structure, (c) Projected density of states (pDOS) of **L1-5** Mo_6_S_3_I_6_ nanowire


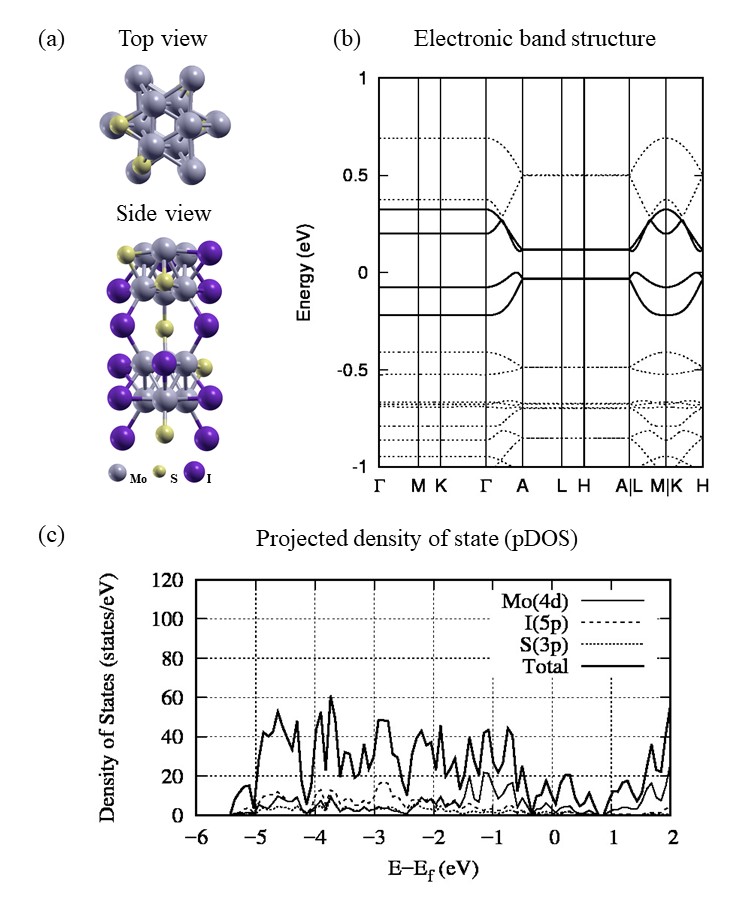


**Figure. S23** Ball and stick atomic structure (Top and Side view), (b) Electronic band structure, (c) Projected density of states (pDOS) of **L1-6** Mo_6_S_3_I_6_ nanowire


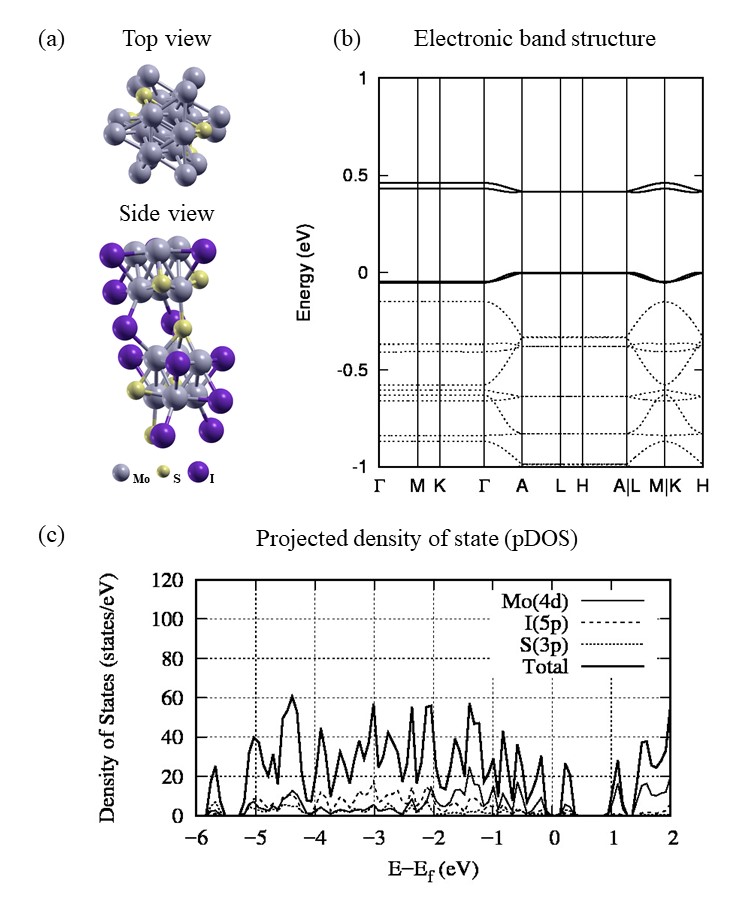


**Figure. S24** Ball and stick atomic structure (Top and Side view), (b) Electronic band structure, (c) Projected density of states (pDOS) of **L1-7** Mo_6_S_3_I_6_ nanowire


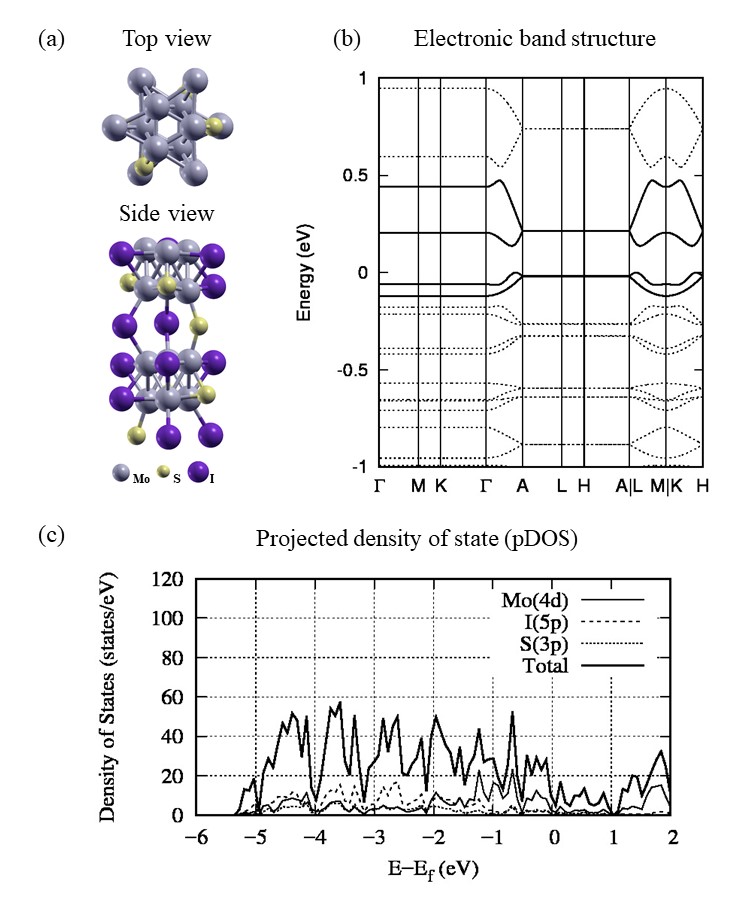


**Figure. S25** Ball and stick atomic structure (Top and Side view), (b) Electronic band structure, (c) Projected density of states (pDOS) of **L1-8** Mo_6_S_3_I_6_ nanowire


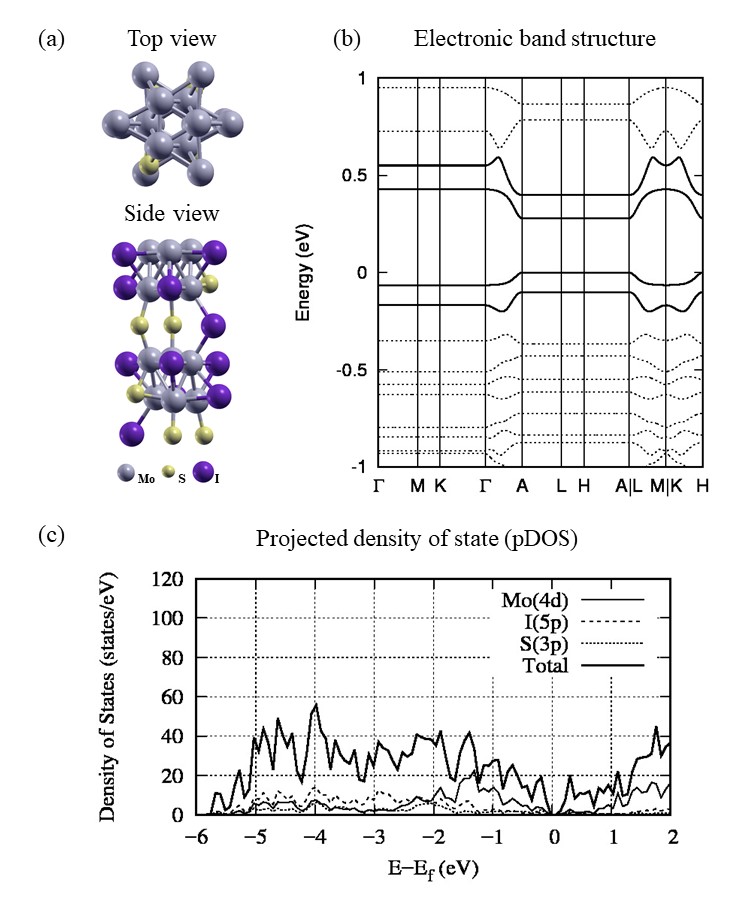


**Figure. S26** Ball and stick atomic structure (Top and Side view), (b) Electronic band structure, (c) Projected density of states (pDOS) of **L2-1** Mo_6_S_3_I_6_ nanowire


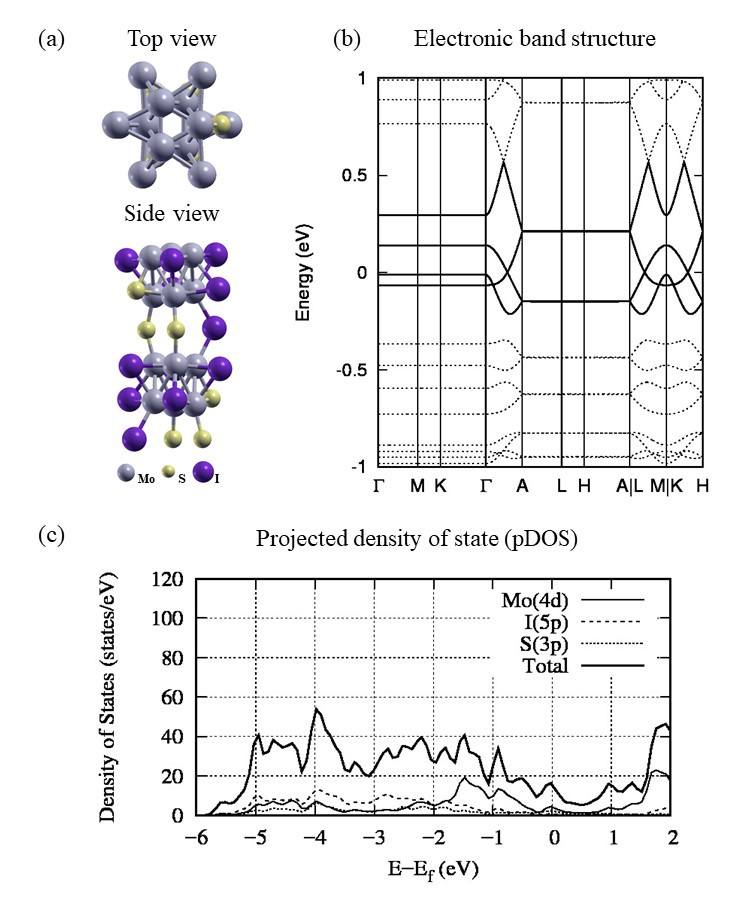


**Figure. S27** Ball and stick atomic structure (Top and Side view), (b) Electronic band structure, (c) Projected density of states (pDOS) of **L2-2** Mo_6_S_3_I_6_ nanowire


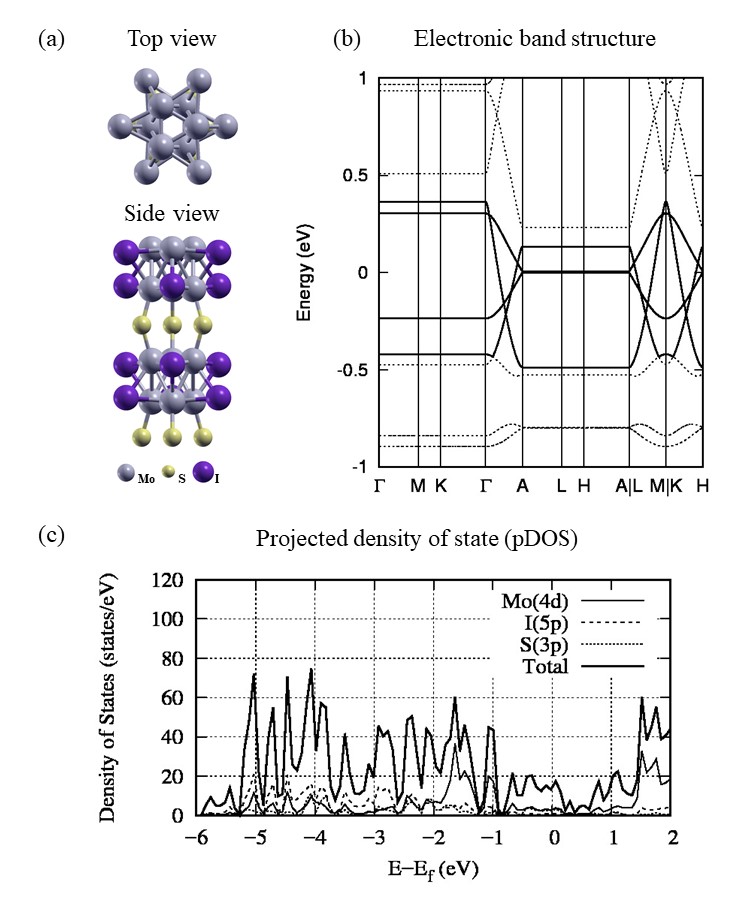


**Figure. S28** Ball and stick atomic structure (Top and Side view), (b) Electronic band structure, (c) Projected density of states (pDOS) of **L3-1** Mo_6_S_3_I_6_ nanowire


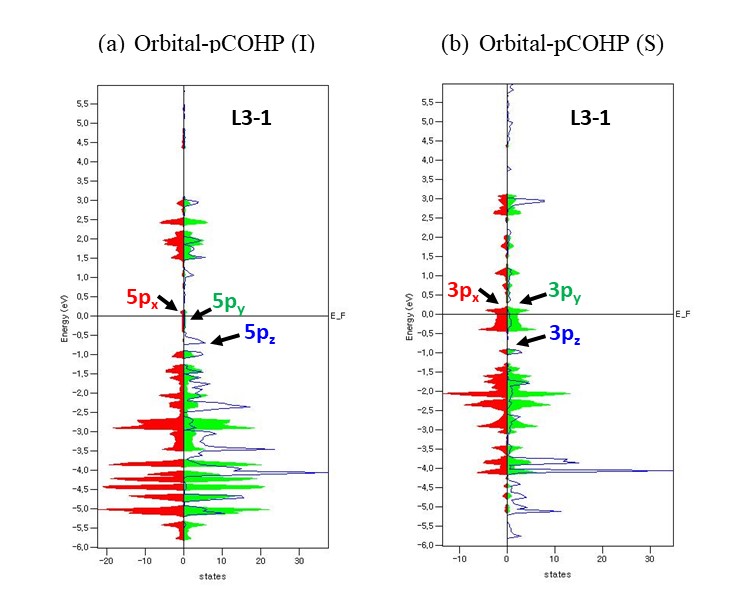


**Figure. S29** The orbital-pDOS of (a) I-5p, and (b) S-3p for L3-1 conformer of a Mo_6_S_3_I_6_ nanowire. The filled red, filled green, and blue lines denote the contribution of p_x_, p_y_, and p_z_ orbital, respectively. The main contributions of sulfur 3p_x_ and 3p_z_ orbital are shown near the Fermi level for conductor, as explained in the main text. The p_x_ orbital energies of sulfur and iodine atoms are plotted with sign flips for better comparison. The energy axis is shown relative to the Fermi level.


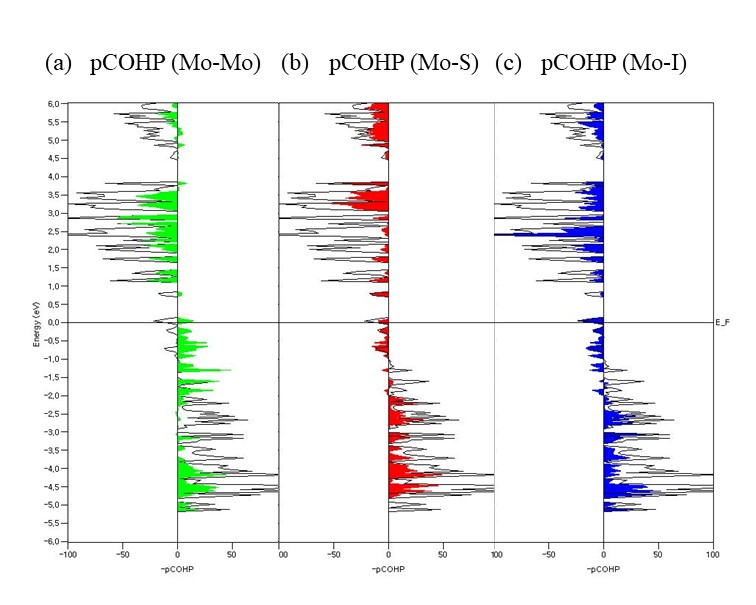


**Figure. S30** Projected crystal orbital Hamilton population (pCOHP) of **S0-1** Mo_6_S_3_I_6_ nanowire for (a) Mo-Mo atom-pair interaction (green), (b) Mo-S atom-pair interaction (red), (c) Mo-I atom-pair interaction (blue). The black solid lines represent the total atom-pair interactions.


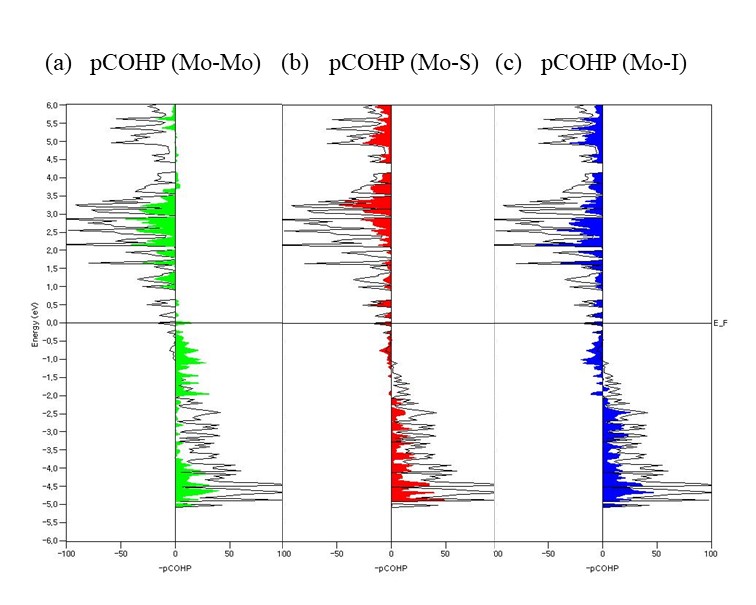


**Figure. S31** Projected crystal orbital Hamilton population (pCOHP) of **S0-2** Mo_6_S_3_I_6_ nanowire for (a) Mo-Mo atom-pair interaction (green), (b) Mo-S atom-pair interaction (red), (c) Mo-I atom-pair interaction (blue). The black solid lines represent the total atom-pair interactions.


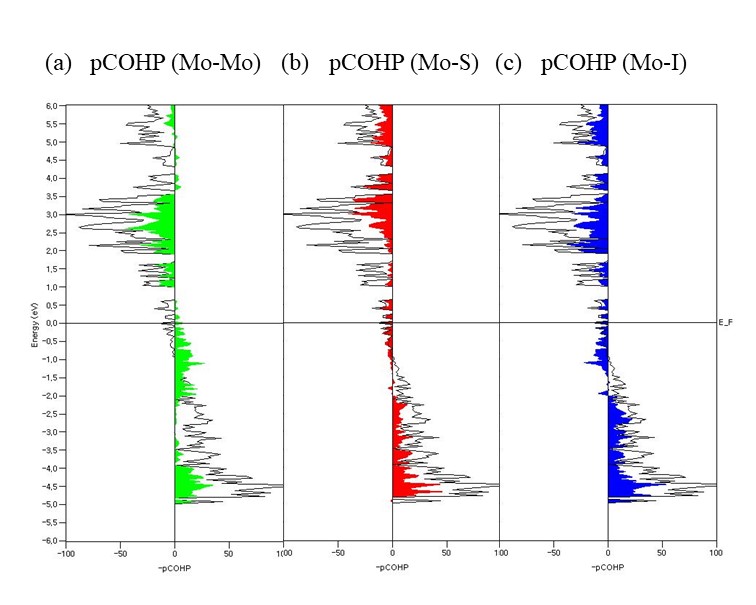


**Figure. S32** Projected crystal orbital Hamilton population (pCOHP) of **S0-3** Mo_6_S_3_I_6_ nanowire for (a) Mo-Mo atom-pair interaction (green), (b) Mo-S atom-pair interaction (red), (c) Mo-I atom-pair interaction (blue). The black solid lines represent the total atom-pair interactions.


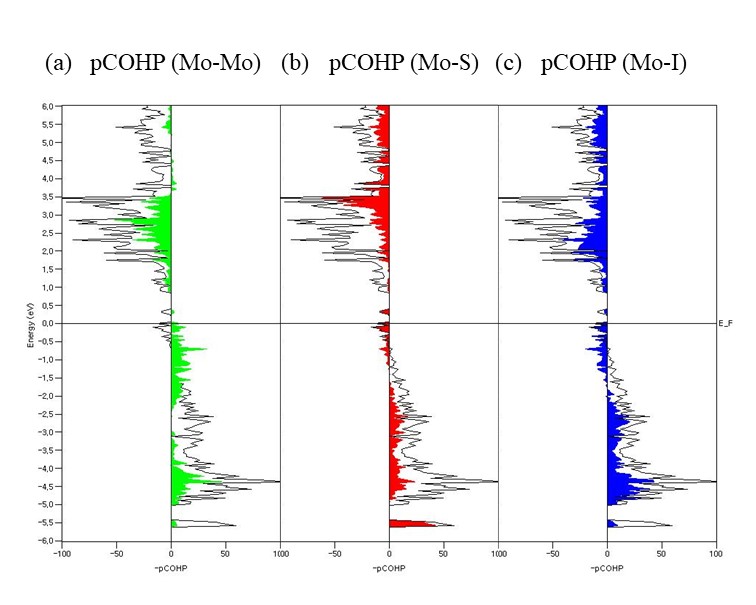


**Figure. S33** Projected crystal orbital Hamilton population (pCOHP) of **S1-1** Mo_6_S_3_I_6_ nanowire for (a) Mo-Mo atom-pair interaction (green), (b) Mo-S atom-pair interaction (red), (c) Mo-I atom-pair interaction (blue). The black solid lines represent the total atom-pair interactions.


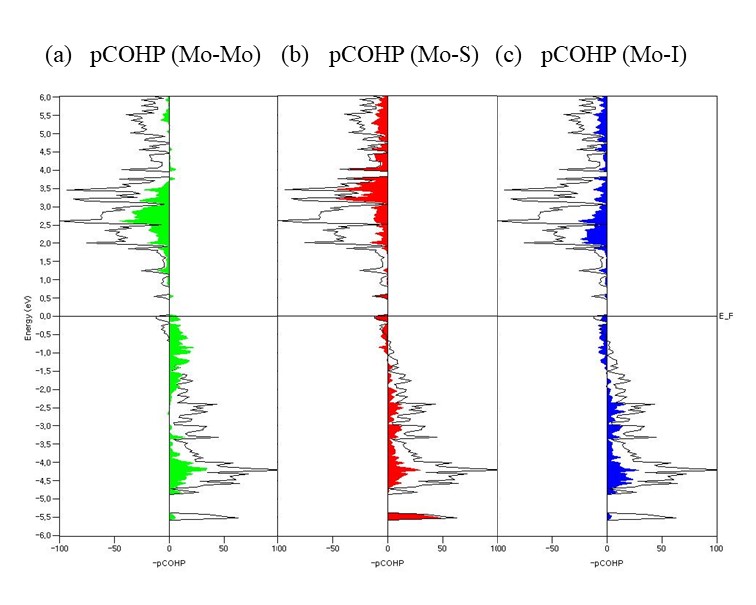


**Figure. S34** Projected crystal orbital Hamilton population (pCOHP) of **S1-2** Mo_6_S_3_I_6_ nanowire for (a) Mo-Mo atom-pair interaction (green), (b) Mo-S atom-pair interaction (red), (c) Mo-I atom-pair interaction (blue). The black solid lines represent the total atom-pair interactions.


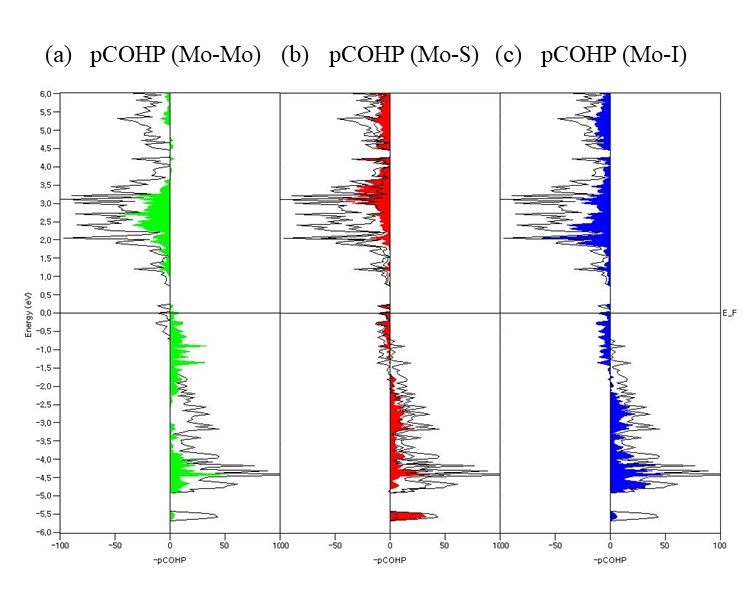


**Figure. S35** Projected crystal orbital Hamilton population (pCOHP) of **S1-3** Mo_6_S_3_I_6_ nanowire for (a) Mo-Mo atom-pair interaction (green), (b) Mo-S atom-pair interaction (red), (c) Mo-I atom-pair interaction (blue). The black solid lines represent the total atom-pair interactions.


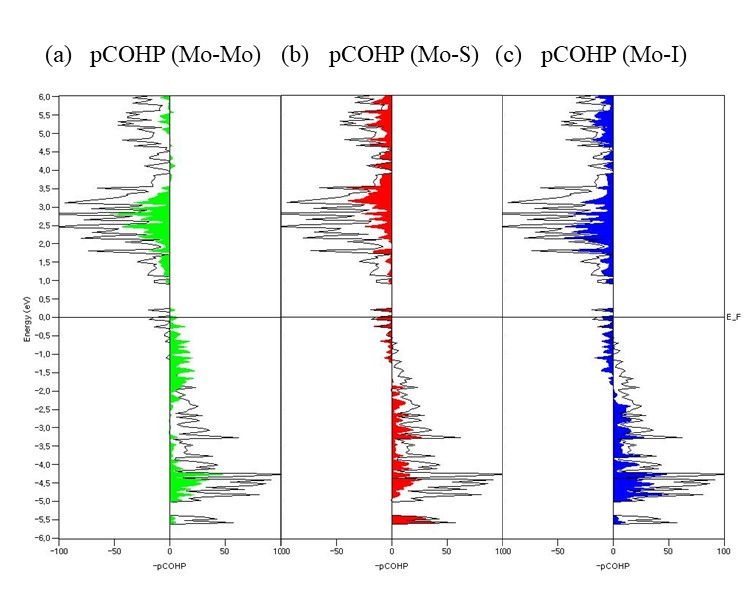


**Figure. S36** Projected crystal orbital Hamilton population (pCOHP) of **S1-4** Mo_6_S_3_I_6_ nanowire for (a) Mo-Mo atom-pair interaction (green), (b) Mo-S atom-pair interaction (red), (c) Mo-I atom-pair interaction (blue). The black solid lines represent the total atom-pair interactions.


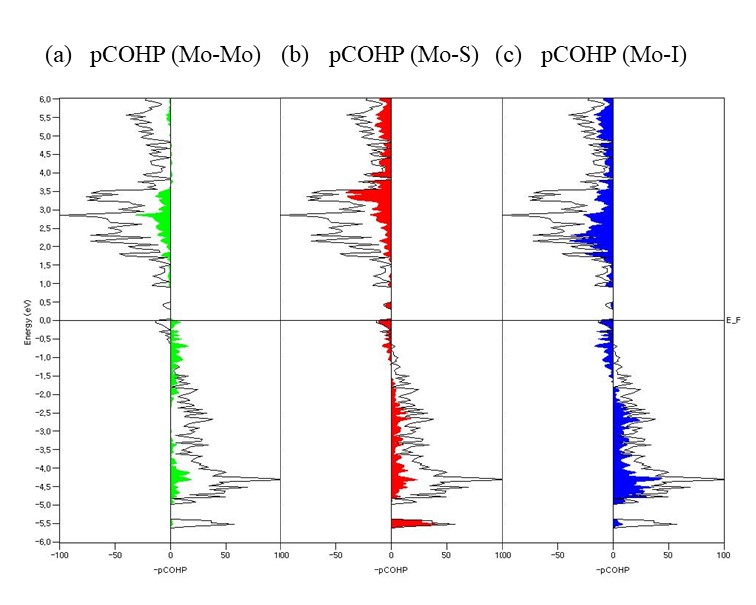


**Figure. S37** Projected crystal orbital Hamilton population (pCOHP) of **S1-5** Mo_6_S_3_I_6_ nanowire for (a) Mo-Mo atom-pair interaction (green), (b) Mo-S atom-pair interaction (red), (c) Mo-I atom-pair interaction (blue). The black solid lines represent the total atom-pair interactions.


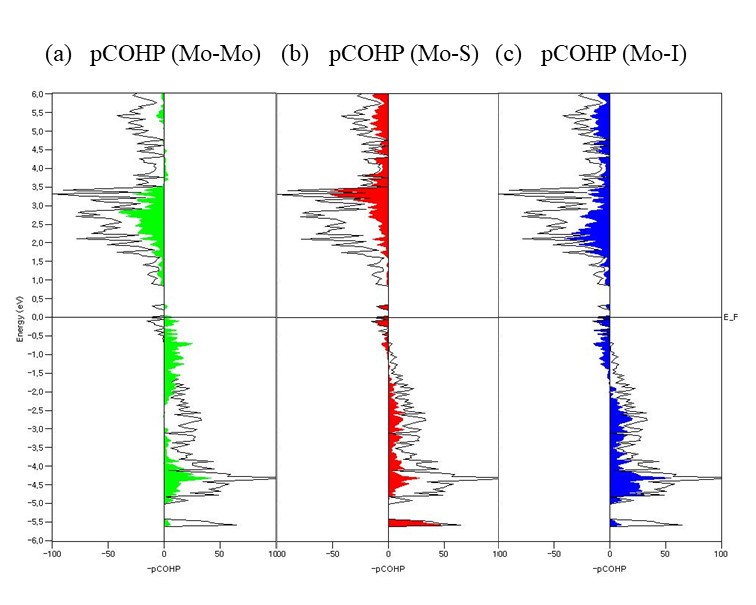


**Figure. S38** Projected crystal orbital Hamilton population (pCOHP) of **S1-6** Mo_6_S_3_I_6_ nanowire for (a) Mo-Mo atom-pair interaction (green), (b) Mo-S atom-pair interaction (red), (c) Mo-I atom-pair interaction (blue). The black solid lines represent the total atom-pair interactions.


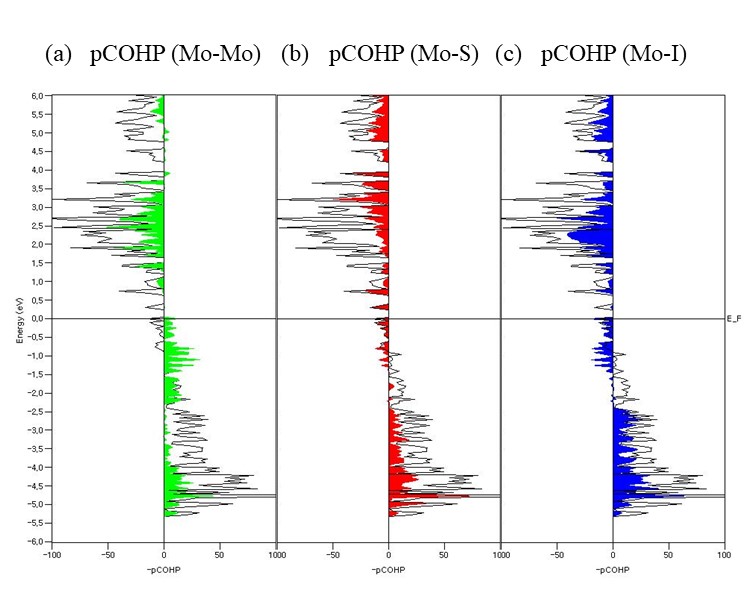


**Figure. S39** Projected crystal orbital Hamilton population (pCOHP) of **S1-7** Mo_6_S_3_I_6_ nanowire for (a) Mo-Mo atom-pair interaction (green), (b) Mo-S atom-pair interaction (red), (c) Mo-I atom-pair interaction (blue). The black solid lines represent the total atom-pair interactions.


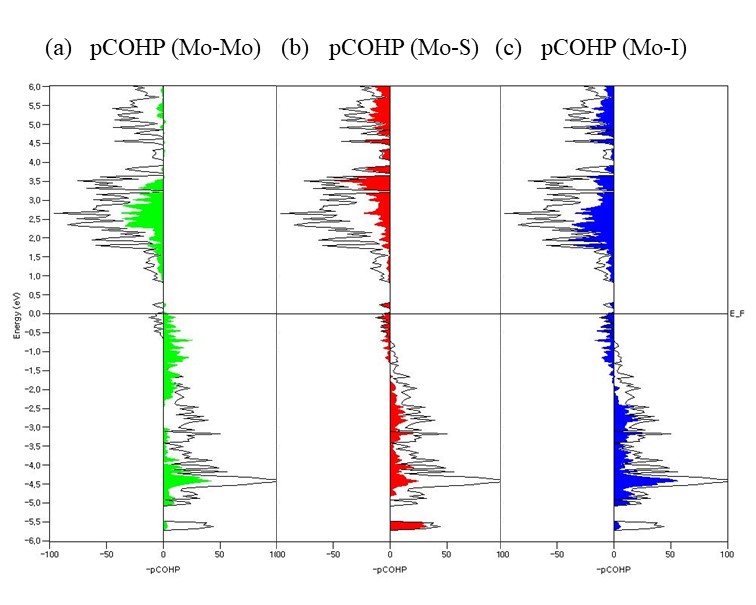


**Figure. S40** Projected crystal orbital Hamilton population (pCOHP) of **S1-8** Mo_6_S_3_I_6_ nanowire for (a) Mo-Mo atom-pair interaction (green), (b) Mo-S atom-pair interaction (red), (c) Mo-I atom-pair interaction (blue). The black solid lines represent the total atom-pair interactions.


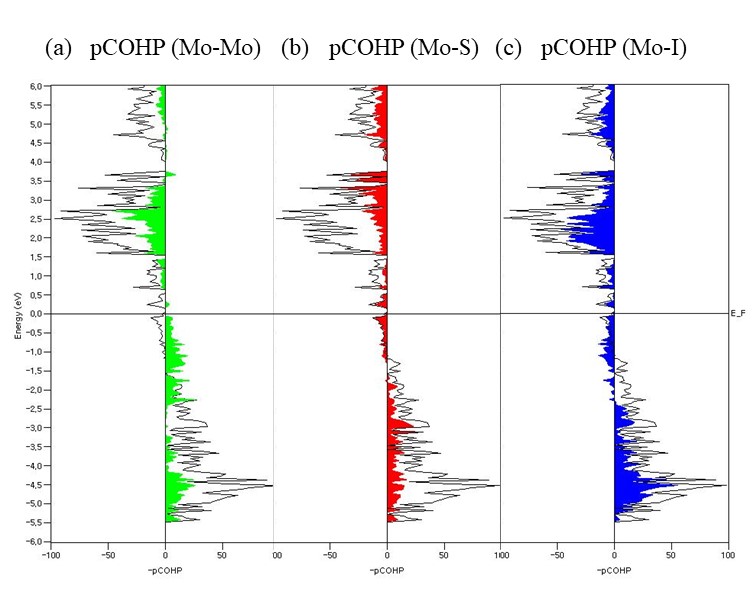


**Figure. S41** Projected crystal orbital Hamilton population (pCOHP) of **S2-1** Mo_6_S_3_I_6_ nanowire for (a) Mo-Mo atom-pair interaction (green), (b) Mo-S atom-pair interaction (red), (c) Mo-I atom-pair interaction (blue). The black solid lines represent the total atom-pair interactions.


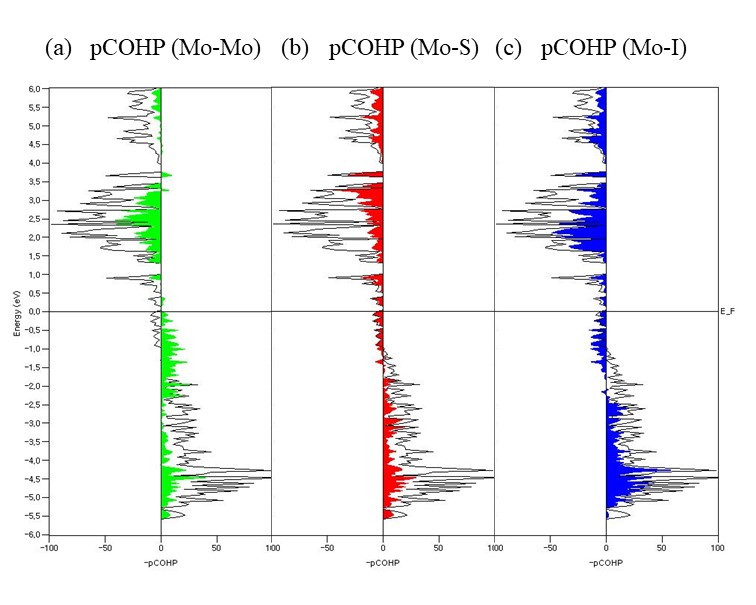


**Figure. S42** Projected crystal orbital Hamilton population (pCOHP) of **S2-2** Mo_6_S_3_I_6_ nanowire for (a) Mo-Mo atom-pair interaction (green), (b) Mo-S atom-pair interaction (red), (c) Mo-I atom-pair interaction (blue). The black solid lines represent the total atom-pair interactions.


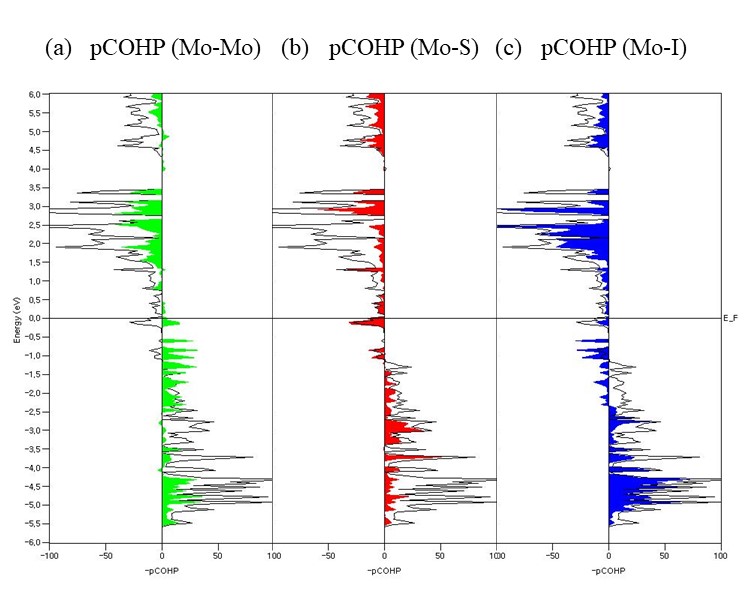


**Figure. S43** Projected crystal orbital Hamilton population (pCOHP) of **S3-1** Mo_6_S_3_I_6_ nanowire for (a) Mo-Mo atom-pair interaction (green), (b) Mo-S atom-pair interaction (red), (c) Mo-I atom-pair interaction (blue). The black solid lines represent the total atom-pair interactions.


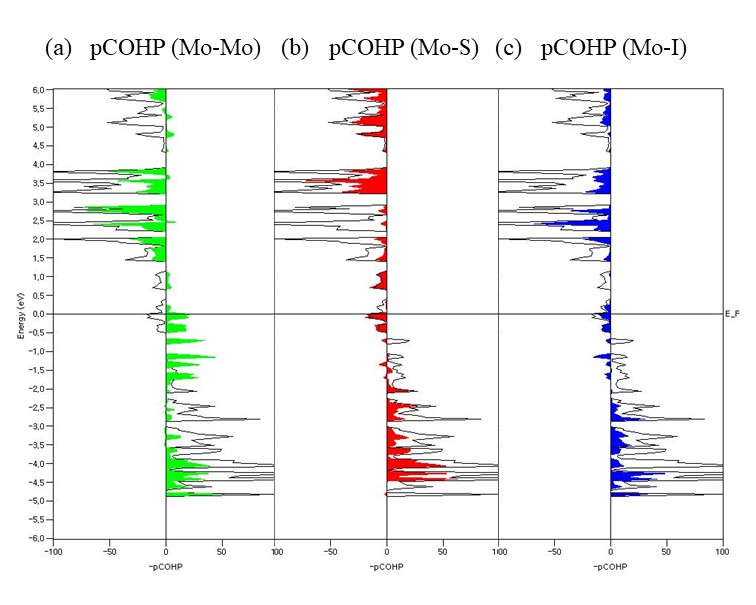


**Figure. S44** Projected crystal orbital Hamilton population (pCOHP) of **L0-1**Mo_6_S_3_I_6_ nanowire for (a) Mo-Mo atom-pair interaction (green), (b) Mo-S atom-pair interaction (red), (c) Mo-I atom-pair interaction (blue). The black solid lines represent the total atom-pair interactions.


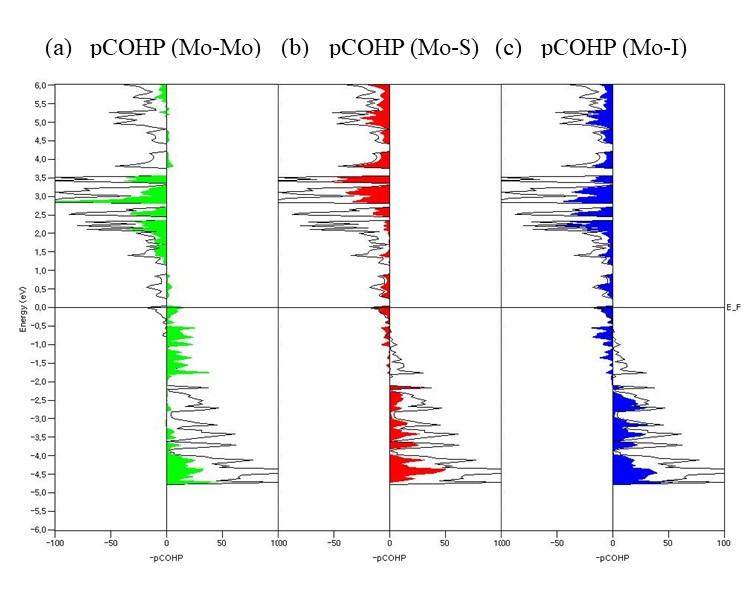


**Figure. S45** Projected crystal orbital Hamilton population (pCOHP) of **L0-2** Mo_6_S_3_I_6_ nanowire for (a) Mo-Mo atom-pair interaction (green), (b) Mo-S atom-pair interaction (red), (c) Mo-I atom-pair interaction (blue). The black solid lines represent the total atom-pair interactions.


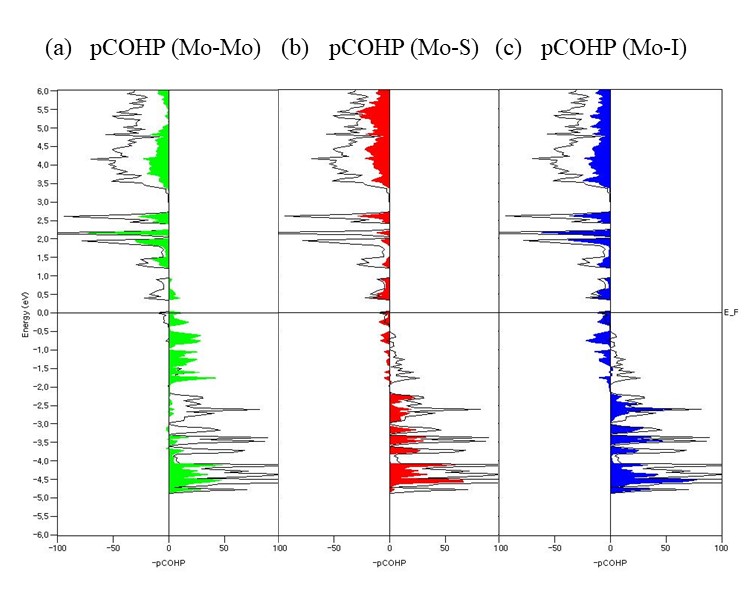


**Figure. S46** Projected crystal orbital Hamilton population (pCOHP) of **L0-3** Mo_6_S_3_I_6_ nanowire for (a) Mo-Mo atom-pair interaction (green), (b) Mo-S atom-pair interaction (red), (c) Mo-I atom-pair interaction (blue). The black solid lines represent the total atom-pair interactions.


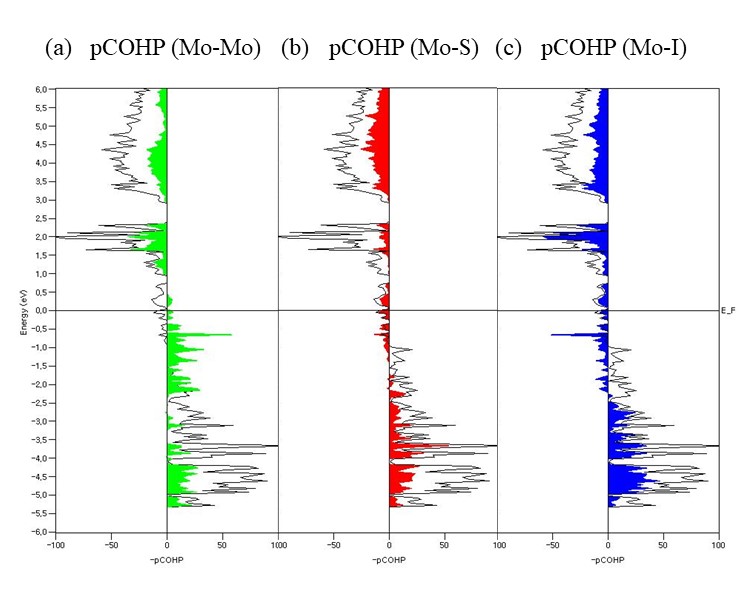


**Figure. S47** Projected crystal orbital Hamilton population (pCOHP) of **L1-1** Mo_6_S_3_I_6_ nanowire for (a) Mo-Mo atom-pair interaction (green), (b) Mo-S atom-pair interaction (red), (c) Mo-I atom-pair interaction (blue). The black solid lines represent the total atom-pair interactions.


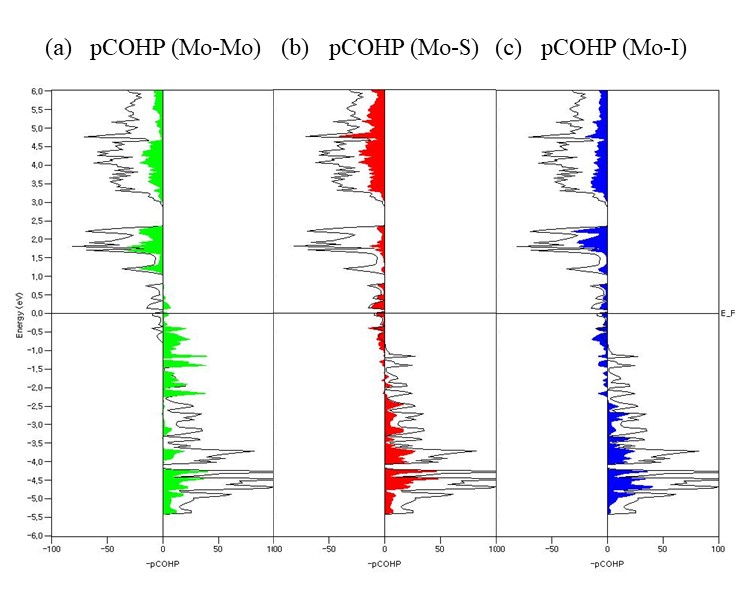


**Figure. S48** Projected crystal orbital Hamilton population (pCOHP) of **L1-2** Mo_6_S_3_I_6_ nanowire for (a) Mo-Mo atom-pair interaction (green), (b) Mo-S atom-pair interaction (red), (c) Mo-I atom-pair interaction (blue). The black solid lines represent the total atom-pair interactions.


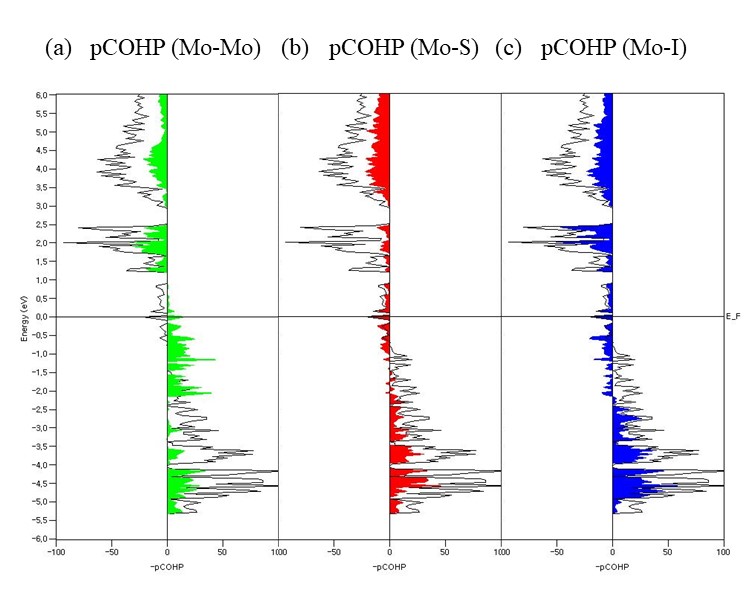


**Figure. S49** Projected crystal orbital Hamilton population (pCOHP) of **L1-3** Mo_6_S_3_I_6_ nanowire for (a) Mo-Mo atom-pair interaction (green), (b) Mo-S atom-pair interaction (red), (c) Mo-I atom-pair interaction (blue). The black solid lines represent the total atom-pair interactions.


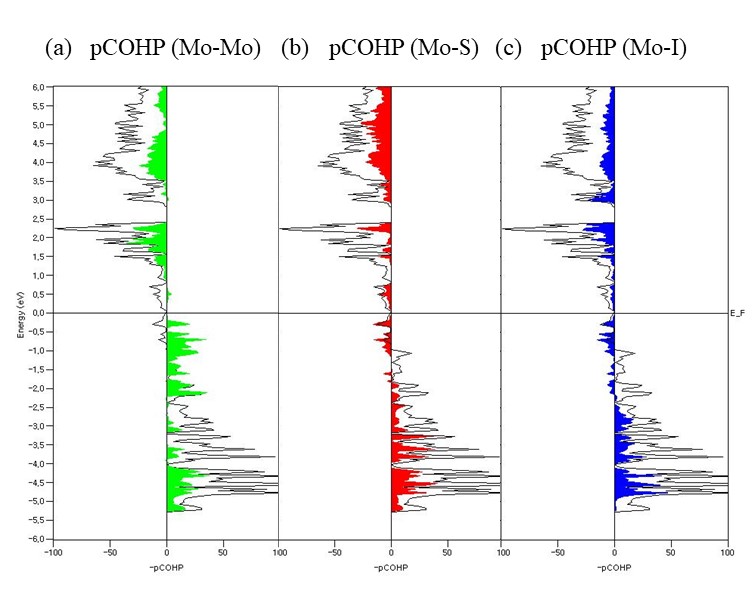


**Figure. S50** Projected crystal orbital Hamilton population (pCOHP) of **L1-4** Mo_6_S_3_I_6_ nanowire for (a) Mo-Mo atom-pair interaction (green), (b) Mo-S atom-pair interaction (red), (c) Mo-I atom-pair interaction (blue). The black solid lines represent the total atom-pair interactions.


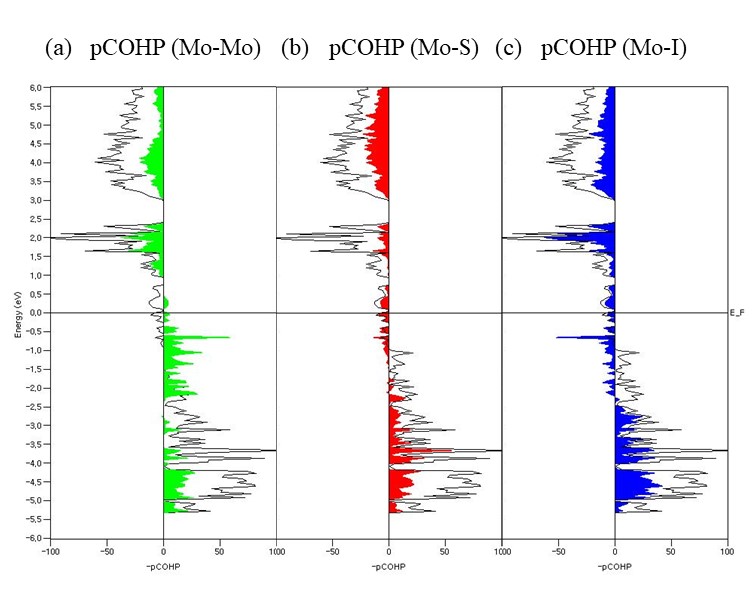


**Figure. S51** Projected crystal orbital Hamilton population (pCOHP) of **L1-5** Mo_6_S_3_I_6_ nanowire for (a) Mo-Mo atom-pair interaction (green), (b) Mo-S atom-pair interaction (red), (c) Mo-I atom-pair interaction (blue). The black solid lines represent the total atom-pair interactions.


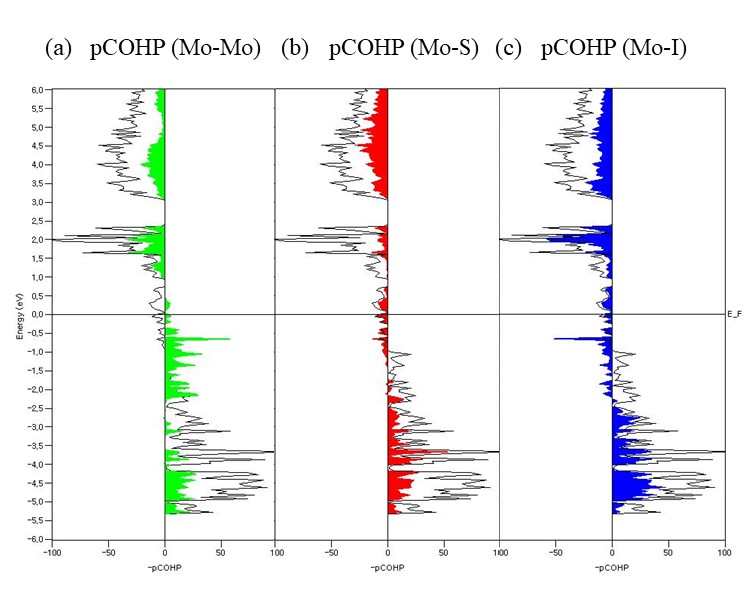


**Figure. S52** Projected crystal orbital Hamilton population (pCOHP) of **L1-6** Mo_6_S_3_I_6_ nanowire for (a) Mo-Mo atom-pair interaction (green), (b) Mo-S atom-pair interaction (red), (c) Mo-I atom-pair interaction (blue). The black solid lines represent the total atom-pair interactions.


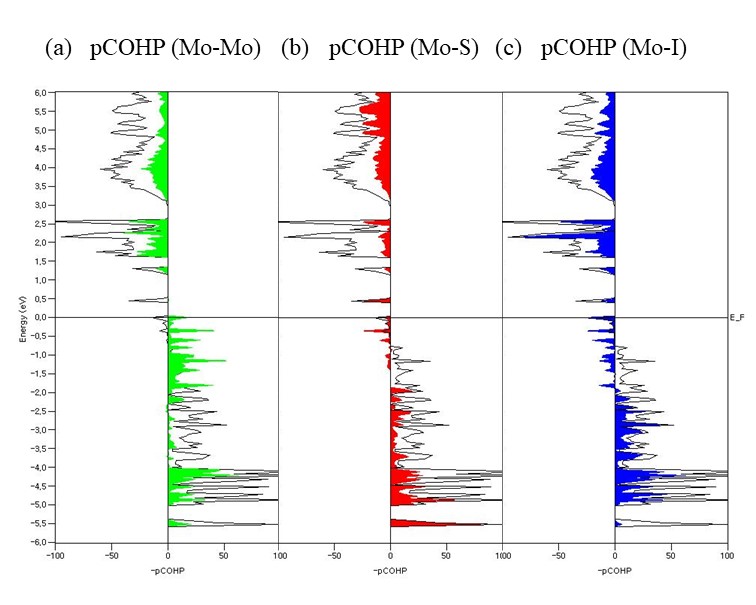


**Figure. S53** Projected crystal orbital Hamilton population (pCOHP) of **L1-7** Mo_6_S_3_I_6_ nanowire for (a) Mo-Mo atom-pair interaction (green), (b) Mo-S atom-pair interaction (red), (c) Mo-I atom-pair interaction (blue). The black solid lines represent the total atom-pair interactions.


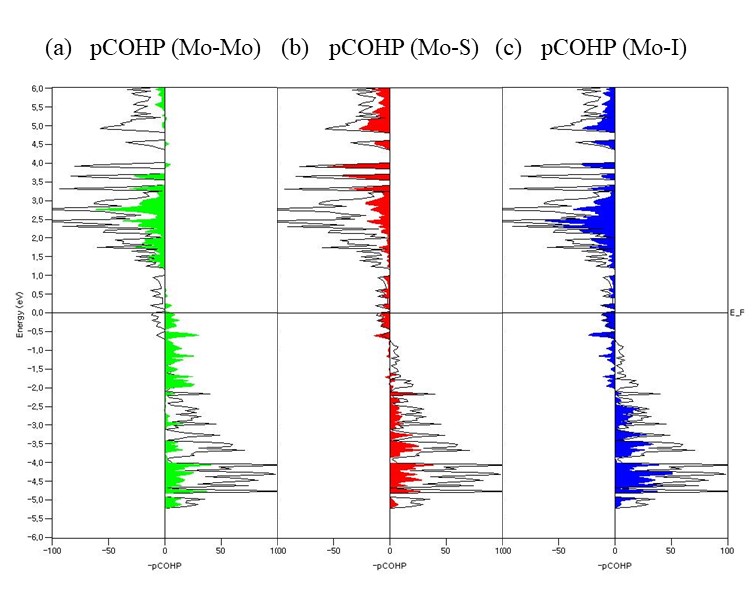


**Figure. S54** Projected crystal orbital Hamilton population (pCOHP) of **L1-8** Mo_6_S_3_I_6_ nanowire for (a) Mo-Mo atom-pair interaction (green), (b) Mo-S atom-pair interaction (red), (c) Mo-I atom-pair interaction (blue). The black solid lines represent the total atom-pair interactions.


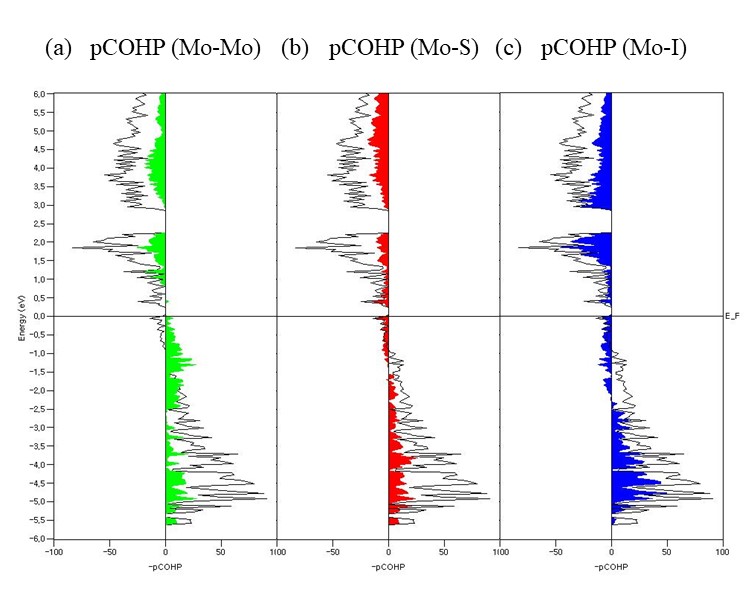


**Figure. S55** Projected crystal orbital Hamilton population (pCOHP) of **L2-1** Mo_6_S_3_I_6_ nanowire for (a) Mo-Mo atom-pair interaction (green), (b) Mo-S atom-pair interaction (red), (c) Mo-I atom-pair interaction (blue). The black solid lines represent the total atom-pair interactions.


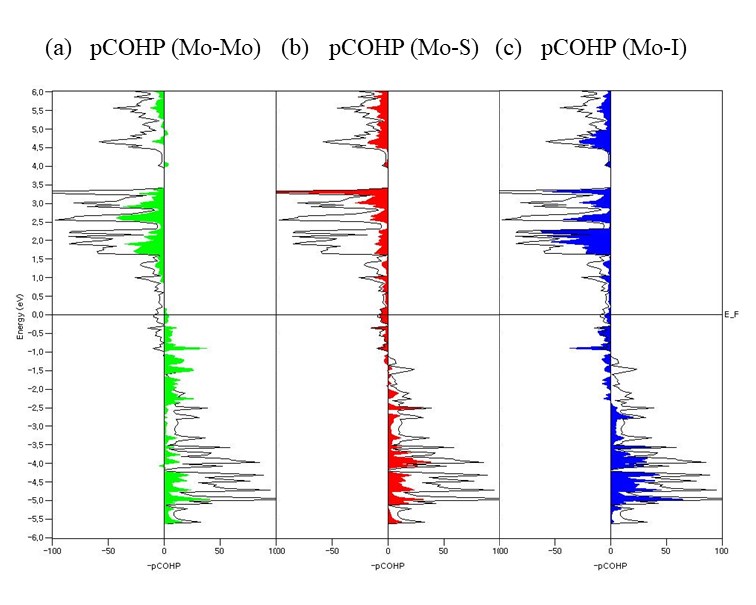


**Figure. S56** Projected crystal orbital Hamilton population (pCOHP) of **L2-2** Mo_6_S_3_I_6_ nanowire for (a) Mo-Mo atom-pair interaction (green), (b) Mo-S atom-pair interaction (red), (c) Mo-I atom-pair interaction (blue). The black solid lines represent the total atom-pair interactions.


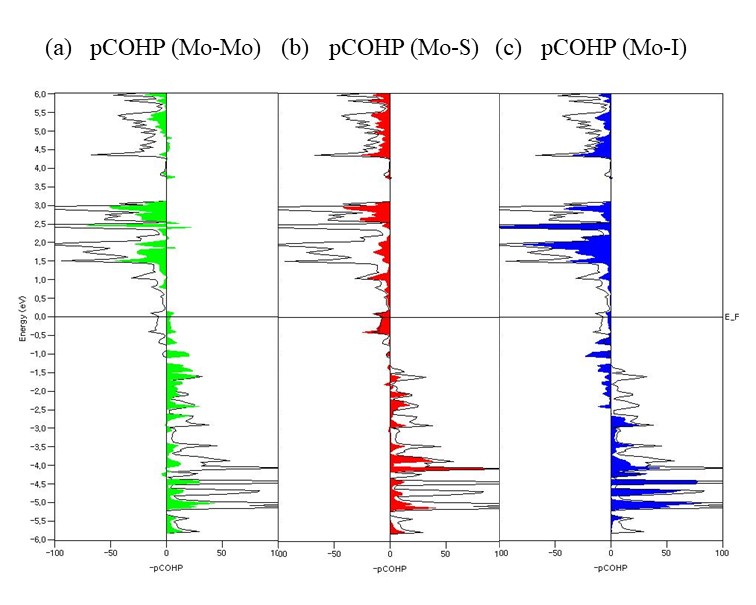


**Figure. S57** Projected crystal orbital Hamilton population (pCOHP) of **L3-0** Mo_6_S_3_I_6_ nanowire for (a) Mo-Mo atom-pair interaction (green), (b) Mo-S atom-pair interaction (red), (c) Mo-I atom-pair interaction (blue). The black solid lines represent the total atom-pair interactions.


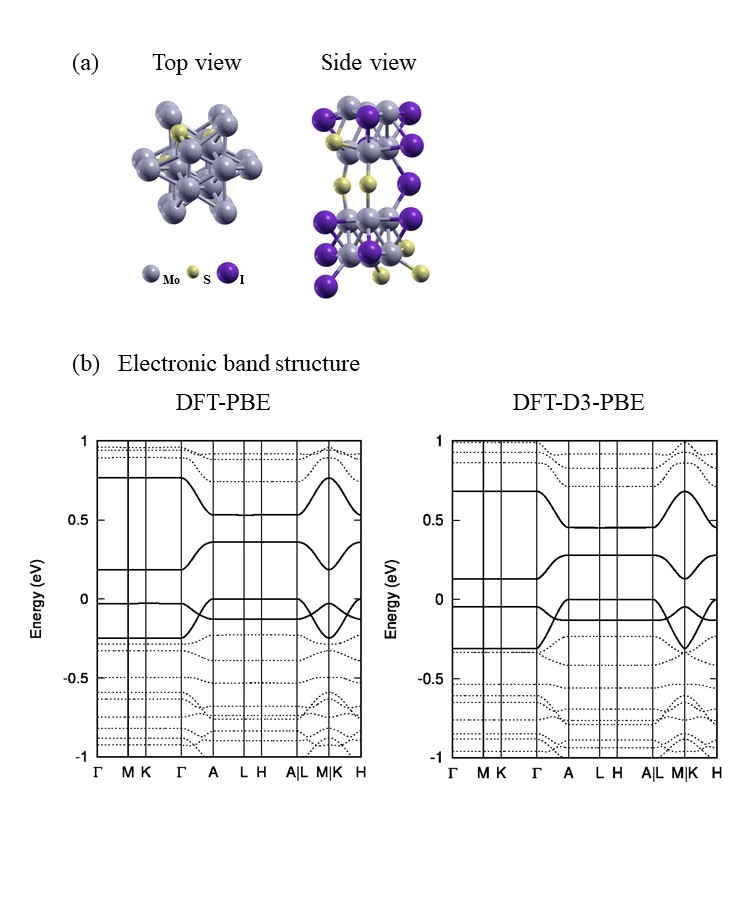


**Figure. S58** The Electronic band structure of **S2-2** Mo_6_S_3_I_6_ nanowire using DFT-D3.


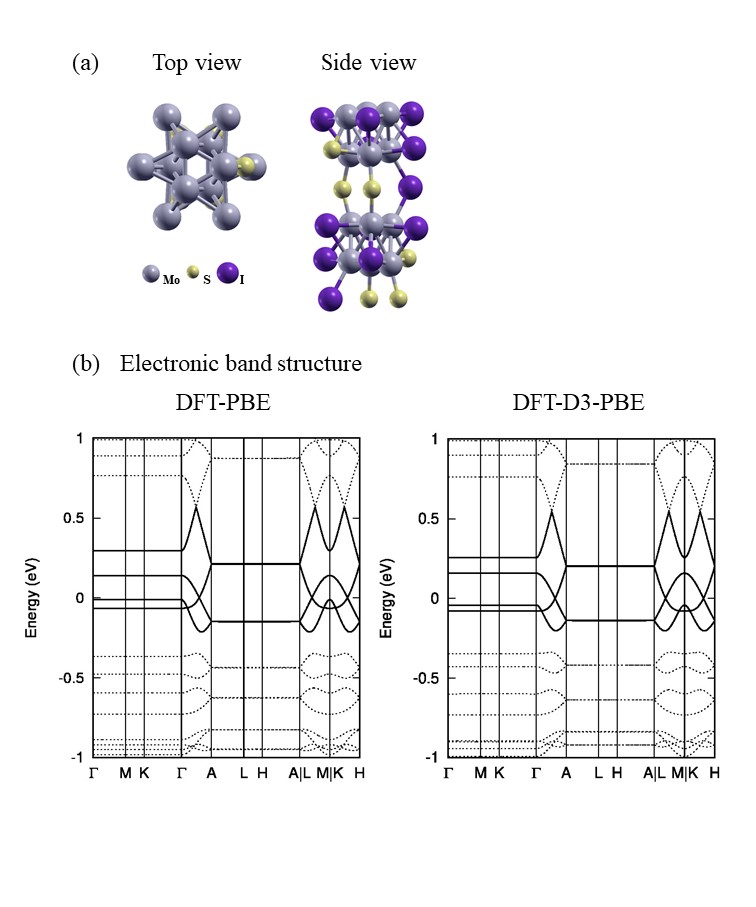


**Figure. S59** The Electronic band structure of **L2-2** Mo_6_S_3_I_6_ nanowire using DFT-D3.

**Table. S1**. The structure parameters such as the optimized equilibrium lattice constants (a, b, and c) and total energy for various models of the short (S) and the long (L) sulfur bridge configurations of Mo_6_S_3_I_6_ nanowires.

| Atomic models | Lattice Constants (Å) | | | | | Total Energy (eV) |
| --- | --- | --- | --- | --- | --- | --- |
|  | a |  | b |  | c |  |
| S0-1 | 15.012 |  | 15.012 |  | 12.521 | -182.24290 |
| S0-2 | 15.025 |  | 15.025 |  | 12.528 | -183.91758 |
| S0-3 | 15.343 |  | 15.277 |  | 11.382 | -183.15185 |
| S1-1 | 15.249 |  | 15.229 |  | 11.634 | -185.04677 |
| S1-2 | 15.262 |  | 15.259 |  | 11.513 | -185.35951 |
| S1-3 | 15.247 |  | 15.169 |  | 11.898 | -184.80867 |
| S1-4 | 15.095 |  | 15.116 |  | 11.834 | -184.86000 |
| S1-5 | 15.164 |  | 15.209 |  | 11.573 | -185.10954 |
| S1-6 | 15.166 |  | 15.211 |  | 11.58 | -185.01760 |
| S1-7 | 14.911 |  | 14.912 |  | 12.773 | -182.26345 |
| S1-8 | 15.180 |  | 15.238 |  | 11.790 | -184.89578 |
| S2-1 | 14.983 |  | 14.983 |  | 12.597 | -181.05858 |
| S2-2 | 15.014 |  | 15.012 |  | 12.442 | -181.38275 |
| S3-1 | 14.974 |  | 14.974 |  | 12.541 | -179.71090 |
|  |  |  |  |  |  |  |
| L0-1 | 15.104 |  | 15.097 |  | 13.47 | -181.60795 |
| L0-2 | 15.082 |  | 15.061 |  | 13.553 | -182.21544 |
| L0-3 | 15.021 |  | 15.021 |  | 13.723 | -182.36974 |
| L1-1 | 15.022 |  | 15.021 |  | 13.761 | -181.29482 |
| L1-2 | 14.963 |  | 14.964 |  | 13.867 | -181.32078 |
| L1-3 | 14.986 |  | 14.988 |  | 13.803 | -184.80607 |
| L1-4 | 15.146 |  | 15.146 |  | 13.518 | -181.76827 |
| L1-5 | 15.020 |  | 15.022 |  | 13.760 | -181.29467 |
| L1-6 | 15.022 |  | 15.021 |  | 13.761 | -181.29451 |
| L1-7 | 15.165 |  | 15.165 |  | 13.197 | -184.07264 |
| L1-8 | 15.015 |  | 15.021 |  | 13.772 | -181.27233 |
| L2-1 | 15.019 |  | 15.017 |  | 13.792 | -180.84520 |
| L2-2 | 14.929 |  | 14.949 |  | 13.902 | -180.76797 |
| L3-1 | 15.031 |  | 15.031 |  | 13.715 | -179.69056 |
